# Establishing *Saccharomyces cerevisiae* as a host for renewable acrylic acid production

**DOI:** 10.1186/s12934-026-02974-3

**Published:** 2026-03-04

**Authors:** Leon Eisentraut, Xiaowei Li, Yun Chen

**Affiliations:** 1 Department of Life Sciences, Chalmers University of Technology, Gothenburg, Sweden; 2 Tianjin Institute of Industrial Biotechnology, Chinese Academy of Sciences, Tianjin, China; 3 Novo Nordisk Foundation Center for Biosustainability, Technical University of Denmark, Kongens Lyngby, Denmark

**Keywords:** Adaptive laboratory evolution, Acrylic acid, Acetyl-CoA transferase, Metabolic engineering, Yeast

## Abstract

Acrylic acid (AA) is an important industrial chemical with a wide range of applications and growing market demand. To explore sustainable production routes, we investigated *Saccharomyces cerevisiae* as a host organism for direct fermentative production of AA. We first assessed the native tolerance of the host to AA and employed adaptive laboratory evolution to obtain strains with improved phenotypes. Whole-genome sequencing of evolved mutants showed that tolerance is linked to the mitochondrial genes *ACH1* and *ETR1*, implicating the prevention of toxic intermediate AA-CoA accumulation as a key tolerance mechanism. While the wildtype strain was able to degrade AA via *ACH1*, a respective knockout mostly abolished degradation. Next, we screened three biosynthetic pathways and identified the β-alanine route as the most promising for direct fermentative production of AA. Finally, batch fermentation in shake flasks yielded titres of 30 mg L⁻¹ AA. These findings provide new insights into the genetic basis of AA tolerance in yeast and establish a foundation for developing *S. cerevisiae* as a platform for renewable AA production.

**Supplementary Information:**

The online version contains supplementary material available at 10.1186/s12934-026-02974-3.

## Introduction

Acrylic acid (AA) is a valuable platform chemical with diverse applications ranging from detergents, absorbents, textiles to colourants and lacks, with an estimated market size of at least $18 billion by 2033 [1, 2]. Its current production method is almost entirely based on petrol oil and has significant emissions of Greenhouse gases as well as industrial waste side streams like toxic acrolein [3, 4]. With chemical producers’ committing to cut down their carbon footprint, eyes have recently been set on renewable, biobased production processes of this important platform chemical [5, 6]. So far, processes on renewable AA have mainly been going via the intermediate 3-hydroxypropionic acid (3HP) with subsequent chemical dehydration. However, to date these processes have yet to prove their economic viability due to the additional chemical catalysis and the further required purification steps increasing costs [6–8].

To overcome the challenge of additional chemical catalysis, the development of a direct fermentation route to AA has recently gained more attention [9–11]. Yet, for such processes to be economically competitive, both cost-effective raw materials and efficient purification strategies must be employed [8, 9]. One strategy is low pH fermentation, which facilitates downstream separation as many organic acids are easier to purify in their respective acid form [12]. A cheap raw materials on the other hand, could be lignocellulosic biomass, which is typically hydrolysed under acidic conditions [13], making low-pH fermentation particularly advantageous. The model yeast *Saccharomyces cerevisiae*, which is known for its intrinsic low-pH tolerance, has already been extensively engineered to withstand the harsh conditions of lignocellulosic hydrolysate, making it a potential host for direct fermentative production of AA [14–16]. Additionally, AA is a highly electrophilic metabolite that disrupts cellular functions through alkylation, exhibiting significant toxicity to cells. This property explains the scarcity of microbial strains capable of naturally synthesizing AA. Previous studies have demonstrated that supplementing the culture medium with AA inhibits the growth of wild-type *Escherichia coli* W3110. Experimental results revealed a progressive reduction in growth rate from 6% to 96% as AA concentration increased from 1.5 mM to 7.5 mM [10]. *S. cerevisiae* is generally known to exhibit notable tolerance to various toxic compounds, including heavy metal ions and organic solvents. Its cell membrane is enriched with sterols such as ergosterol, which enhance membrane stability and provide resistance against the disruptive effects of weak organic acids [17, 18]. However, the tolerance of *S. cerevisiae* towards AA has not yet been systematically characterised.

**Fig. 1:**
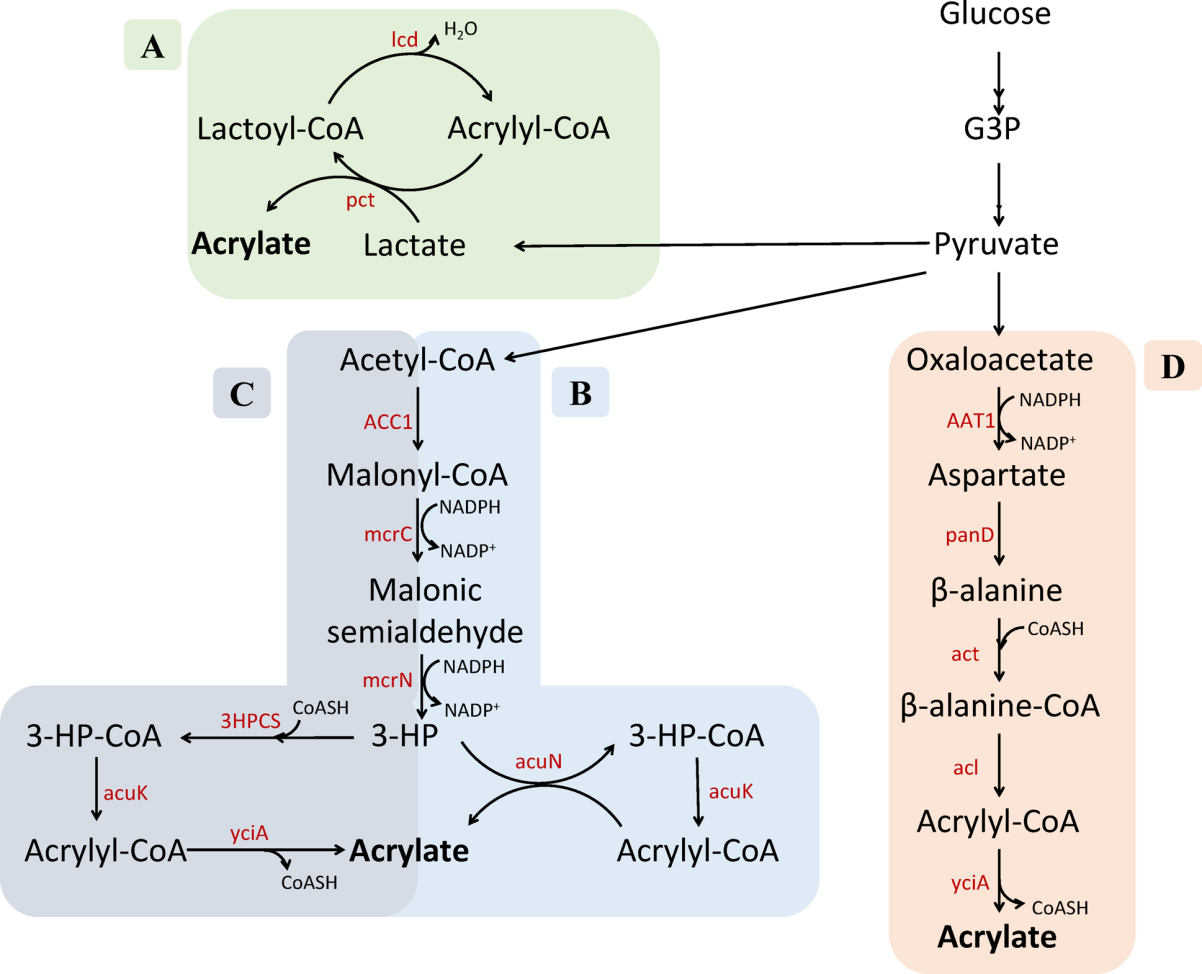
Biosynthetic pathways for direct production of acrylic acid from glucose. **A** Lactate-based pathway from *C. propionicum*, which bypasses the reduction to propionate. **B** 3HP pathway utilizing a CoA transferase system from Halomonas sp. for acrylic acid conversion. **C** 3HP pathway relying on separate enzymatic steps converting 3HP to acrylic acid. **D** Direct fermentation route via β-alanine. Abbreviations: *G3P* Glyceraldehyde-3-phosphate, *3HP* 3-hydroxypropionate, *pct* propionyl-CoA transferase, *lcd* lactoyl-CoA dehydratase, *ACC1* acetyl-CoA carboxylase, *mcrC* malonyl-CoA reductase, *mcrN* malonate semialdehyde reductase, *3HPCS* 3-hydroxypropionate-CoA synthetase, *acuK* acryloyl-CoA hydratase, *yciA* acyl-CoA thioester hydrolase,* acuN* acryloyl-CoA:3-hydroxypropionate CoA-transferase, *AAT1* aspartate aminotransferase, *panD* aspartate 1-decarboxylase, *act* β-alanine CoA transferase, *acl* β-alanyl-CoA: ammonia lyase

Previous research on direct fermentative production of AA showed that AA can be produced from 3HP via 3HP-CoA and AA-CoA and multiple studies have engineered *E. coli* to produce AA via this route [8, 19, 20] (Fig. 1C). Similarly, a CoA-transferase from *Halomonas* sp., a dimethylsulfoniopropionate (DMSP) fermenting bacterium, has been shown to catalyse the interconversion of 3HP and AA-CoA, offering another route to AA production [21] (Fig. 1B). In contrast, alternative pathways bypassing 3HP have also been explored. For instance, the β-alanine route, which avoids conversion to 3HP, has achieved the highest reported AA yield to date in *E. coli* [10] (Fig. 1D). Similarly, Anaerotignum *propionicum (former Clostridium propionicum)*, a lactate-fermenting bacterium, harbours enzymes for converting lactate to AA-CoA, forming yet another potential route [22, 23] (Fig. 1A). However, to our knowledge, no studies have established a direct pathway for AA production in *S. cerevisiae*.

In this study, we applied adaptive laboratory evolution (ALE) to enhance the yeast’s tolerance to AA, together with genome sequencing and reverse engineering we identified modifications that would increase its tolerance towards AA. Furthermore, we evaluated three biosynthetic pathways to identify the most promising route for further development.

## Results and discussion

### Evaluating the tolerance of *S. cerevisiae* to acrylic acid

We first tested the tolerance of *S. cerevisiae* to AA at different pH levels by measuring growth in medium with AA concentrations ranging from 50 to 1000 mg L^− 1^. Our results revealed a strong inhibitory effect of AA on the ethanol phase at pH 6.5, hindering growth from 300 mg L^− 1^ and abolishing it above 400 mg L^− 1^. This effect was more pronounced at pH 6 and pH 5 where ethanol-phase growth was abolished already at lower concentrations. Moreover, at pH 5 and 6 we also discovered an inhibitory effect on the glucose phase with increasing AA concentration (Supplementary Fig. 1). The influence of pH on the toxicity of organic acids is well documented and inhibition of ethanol-phase growth has also been observed with other organic acids like propionic acid [24, 25]. However, AA exhibits a significantly lower inhibitory concentration compared to e.g., propionic acid or acetic acid, which cannot be solely attributed to acidity, as AA has the lowest pKa (4.25) among these acids (acetic acid: 4.76; propionic acid: 4.88) [10, 26]. The increased toxicity is likely due to AA being a highly electrophilic metabolite that disrupts cellular functions through alkylation and it has previously shown to disrupt the fatty acid cycle in *E. coli* [27]. When we analysed the fermentation profile of some of the cultivations, we unexpectedly discovered that AA was rapidly degraded by the wildtype strain at concentrations up to 150 mg L^− 1^ (Supplementary Fig. 2).

### Adaptive laboratory evolution of acrylic acid tolerance

**Fig. 2:**
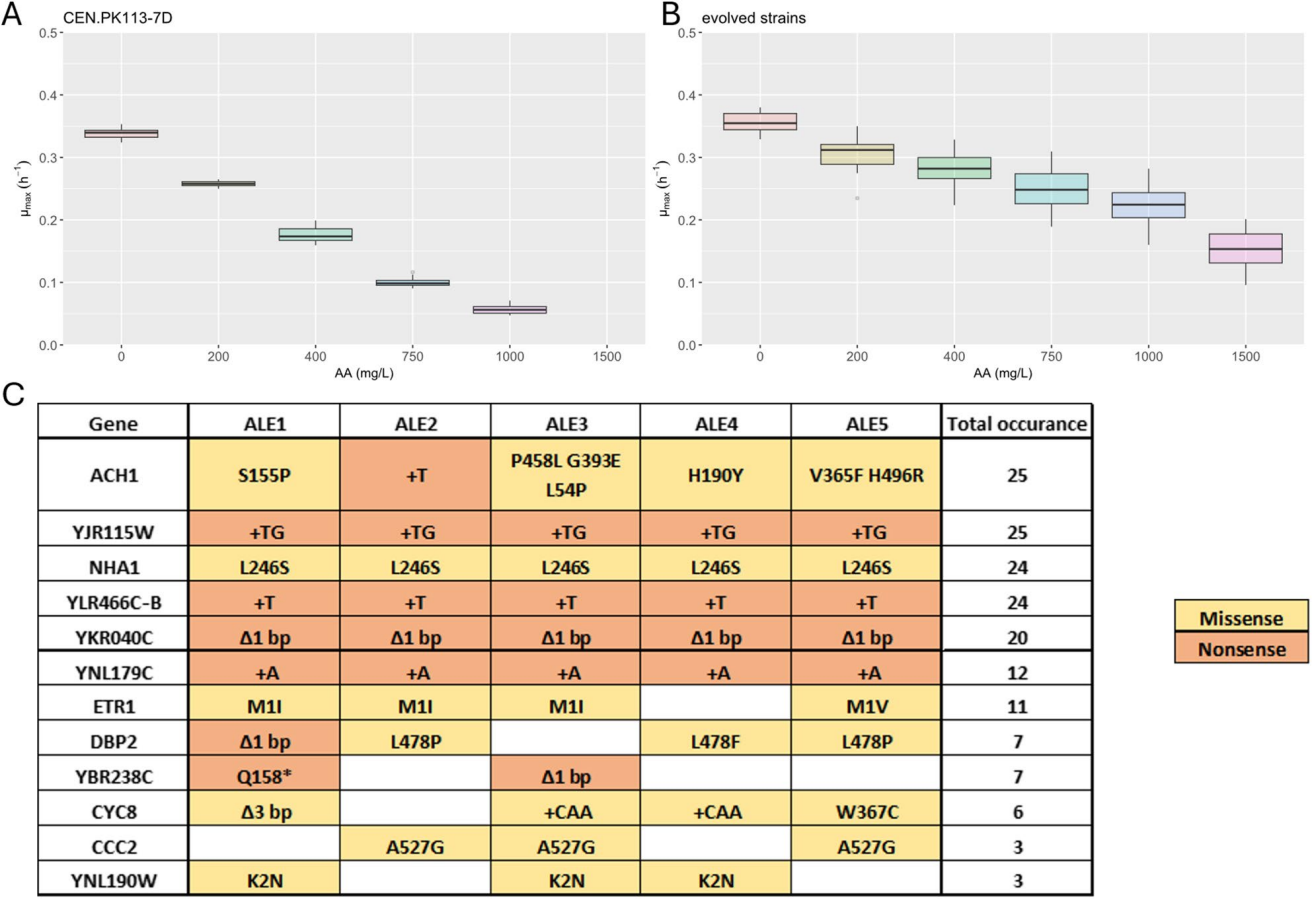
Summary of the results from the adaptive laboratory evolution experiments for tolerance to AA. Distribution of the maximum specific growth rate of the reference strain at different AA concentrations in the evolution media (**A**) and the evolved isolates (**B**). The horizontal line in the box represents the median of the data and the upper and lower edges of the box correspond to the 25th and 75th percentile respectively. The reference represents the data from 4 biological replicates with 3 technical replicates each, while (**B**) includes 26 evolved isolates derived from five independent ALE populations (up to six isolates per population) with 1 technical replicate each. **C** Mutations identified in the same evolved isolates shown in (**B**). Genes mutated in more than one evolution line are shown. Missense mutations (amino acid substitution) and predicted loss-of-function mutations (e.g., frameshift) are indicated by colour coding. The total occurrence is the number of single isolates the respective gene was found mutated in. A detailed list of all mutations can be found in Supplementary Table 1

To specifically select for tolerance against AA while minimizing pH-related effects, we performed evolution at pH 5 using a buffered medium containing 0.15 M citrate-phosphate buffer as previously established [28, 29]. Preliminary growth tests showed that the maximum growth rate of CEN.PK113-7D was nearly halved at 400 mg L^− 1^ (Fig. 2A), which was used as the starting concentration for the ALE. Five different populations (ALE1-5) were initiated from the same preculture and evolved independently for ~50 serial transfers. At a concentration of 1300 mg L^− 1^, cultures transferred at the end of glucose phase had a lag phase of ~24 h upon reinoculation. Notably, only two clones could be recovered from the final culture of ALE4, both showing comparatively lower growth rate than isolates from other populations. All the selected isolates were able to grow at 1500 mg L^− 1^ (Fig. 2B) with some showing tolerance to even higher concentrations (Supplementary Fig. 3), whereas the wildtype could not grow.

Since the wildtype degraded AA at concentrations below 150 mg L^− 1^, we were interested to see if the evolved mutants had evolved to be able to degrade AA at higher concentrations. Interestingly, the concentration of AA remained stable across the conditions (data not shown), indicating that the evolved mutants lost the ability to degrade AA.

### Whole genome sequencing of evolved mutants

To determine the genotype responsible for the AA tolerance, whole genome re-sequencing was performed on the evolved mutant clones (total of 25 clones). Figure 2C shows an overview of the genes mutated in at least two independently evolved populations (full list Supplementary Table 1). However, some of the targets occurring at high frequency all showed an identical mutation (*YJR115W*, *NHA1*, *YLR466C-B*, *YKR040C* and *YNL179C*) suggesting these mutations already occurred in the preculture and are not relevant for the tolerance phenotypes. Additionally, the mutations in *YLR466C-B* and *NHA1* have been previously found in evolutions using the same buffered media (29, unpublished data). Furthermore, mutations in *DBP2*, *CCC2* and *YNL190W*, were supported only by partial reads and were considered unreliable.

Mutations in *ACH1* seem to be of crucial importance given its occurrence in all sequenced clones as well as the diversity of mutations. The fact that all clones in ALE2 had a nonsense mutation, as well as the G393E substitution, which was shown to inactivate the native function of this enzyme as it targets the active site [30], suggested a loss of function was responsible for improved tolerance. ACH1p is a mitochondrial succinyl-CoA: Acetate CoA transferase with additional acetyl-CoA-hydrolase activity that facilitates the export of mitochondrial acetyl-CoA by converting them to acetate [30–32]. Based on its function and broad substrate specificity [31] ACH1p might have an activity transferring a CoA-moiety to acrylate generating acryloyl-CoA which is more toxic than the free acid [33].

The observed mutations in *ETR1* followed an interesting pattern, with three different mutation variants of the start codon detected (ATG → ATT, ATC, or GTG). An analysis of existing literature on *ETR1* indicated that this mutation likely results in the loss of an 8 amino acid long signal peptide responsible for targeting the protein to the mitochondria [34]. The same authors also demonstrated that the absence of the N-terminal signal peptide causes most of the protein to localise to the cytosol yet retaining its activity as an NADPH dependent 2-enoyl thioester reductase and mitochondrial function. As AA is a 2-enoyl acid, mislocalised ETR1p might act on AA-CoA.

Connecting the loss of *YBR238C*, either through frameshift or insertion of a stop codon, to AA tolerance is more difficult than the previously discussed mutations. Little is known about YBR238Cp apart from it being localised to the mitochondria like ACH1p and ETR1p and being involved in mitochondrial function and lifespan [35, 36]. It’s paralog RMD9p regulates mitochondrial gene expression and translation [37–39]. Deletion of *YBR238C* has been associated with altered mitochondrial DNA copy number and increased ATP levels [36], potentially facilitating AA export.

*CYC8* occurred in multiple lineages, consistent with previous reports on tolerance to other organic acids [40] or sodium chloride [41]. The mutations observed in this study were however, mainly in the variable CAA region adding or removing a glutamine therefore not suggesting a prominent effect. One isolate lacking the common *NHA1*^L246S^ mutation carried instead a *CYC8*^W367C^ mutation, suggesting its compensatory role for sodium tolerance. Notably, CYC8p is a transcriptional co-repressor involved in regulation of various genes including *ETR1* [42, 43], linking it to the other mutations in this study.

### Reverse engineering of mutations to evaluate their effect

**Fig. 3:**
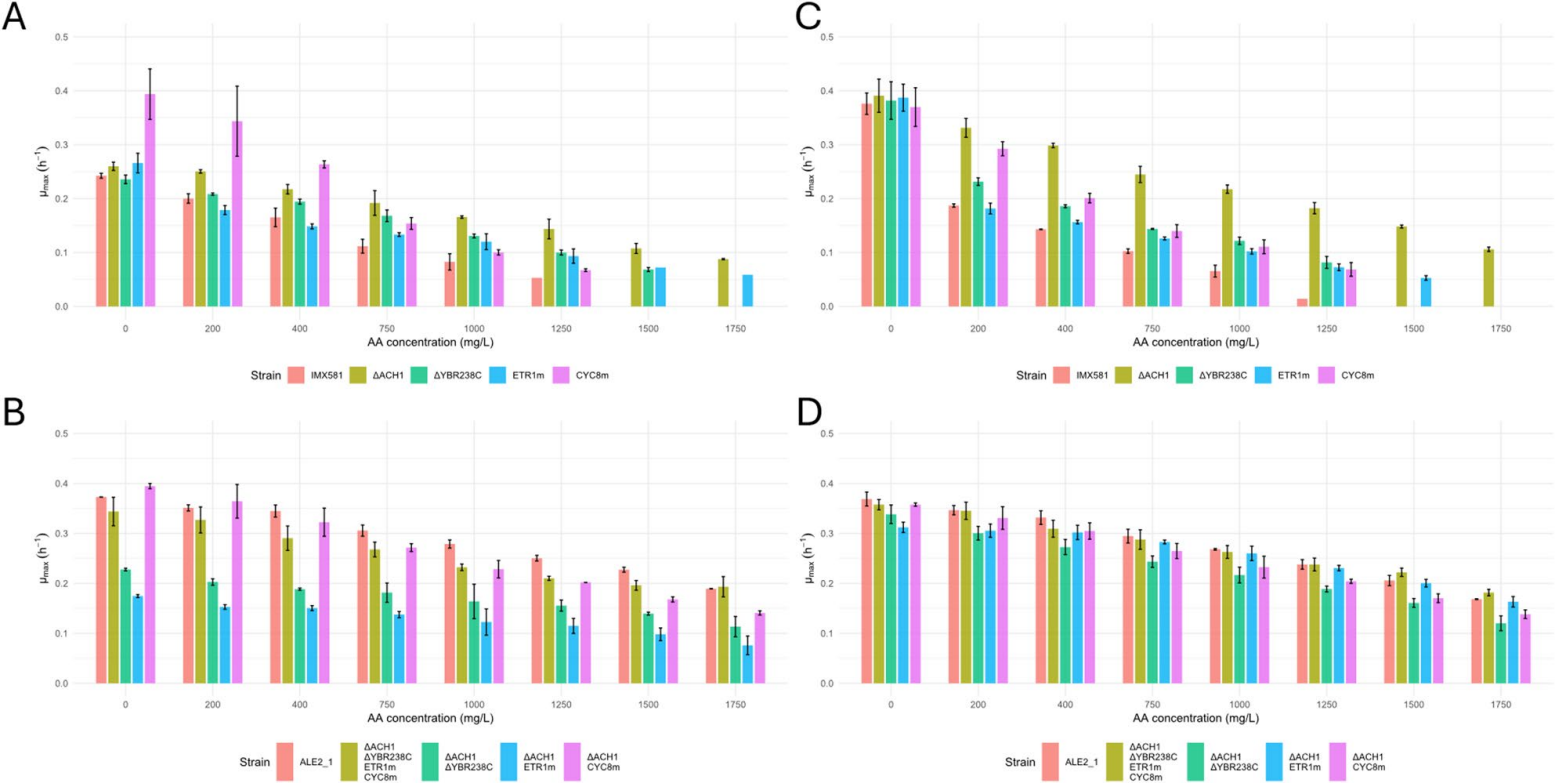
Overview of the maximum specific growth rates achieved by the reverse-engineered strains. **A** Effect of individual mutations at different AA concentrations compared to the reference strain, when grown in sodium buffered Verduyn medium. **B** Cumulative effect of the mutations or just combined with the *ACH1* deletion, compared with one of the best performing evolved clones in sodium buffered Verduyn medium. **C** and **D** – Effect of the same mutations as in (**A**) and (**B**) respectively, when the strains were grown in potassium buffered Verduyn medium. Bar plots represent the mean of *n* = 2 (sodium buffered) and *n* = 3 (potassium buffered) biological replicates, error bars indicate standard deviation

To determine which mutations confer tolerance to AA, we reconstructed the most likely variants individually and the mutations in the resulting strains (*ΔACH1*, *ETR1*^M1V^, *CYC8*^W367C^ and *ΔYBR238C*) were used for further phenotypic characterisation. In the evolution media, *CYC8*^W367C^ lead to a growth rate increase in the absence of AA (Fig. 3A). As the relative growth advantage dropped with increasing AA in the media, we concluded that this mutation primarily confers sodium tolerance rather than AA resistance. By contrast, the *ACH1* deletion showed the strongest positive effect on AA tolerance while the other tested mutations showed moderate improvements. To exclude sodium effect, we repeated the test in a medium exchanging sodium phosphate for potassium phosphate, as CEN.PK background strains are sensitive to sodium [44, 45]. In this medium, all tested mutations improved growth at AA concentrations above 750 mg L^− 1^, with *ΔACH1* again showing the largest effect (Fig. 3).

Nevertheless, none of the strains was able to replicate the full tolerance phenotype of the evolved strains. Therefore, we combined mutations with the *ACH1* deletion to test for synergistic effects. Additional deletion of *YBR238C* or introduction of the *CYC8*^W367C^ allele did not yield a significant increase in growth rate. The *ΔACH1 ETR1*^M1V^ double mutant as well as the strain with 4 mutations fully recovered the tolerance phenotype of the evolved clone (Fig. 3). Lastly, we also tested the combined mutations in the original medium containing sodium. Only the quadruple mutant was able to grow like the evolved ALE isolate (ALE2_1), which served as reference, at high AA concentrations, further indicating that both AA and sodium stress shaped the adaptive response (Fig. 3B).

Since the evolved clones were no longer able to degrade AA, we suspected *ACH1* as the responsible target based on its occurrence and annotation. Other than the WT, the *ΔACH1* strain (LE_AA002) was no longer able to degrade AA at concentrations of 50, 100 and 150 mg L^− 1^ in YPD (data not shown), confirming its role in degradation. With the degradation target identified, we opted to use the *ΔACH1* background as a basis for pathway screening, as deletion of *ACH1* alone resulted in loss of AA degradation and accounted for the largest phenotypic effect under the screening conditions.

### Pathway screening for direct fermentative production of AA

Using the Δ*ACH1* background we were able to screen different pathways and enzyme combinations for their efficiency in direct fermentative production of AA.

**Fig. 4:**
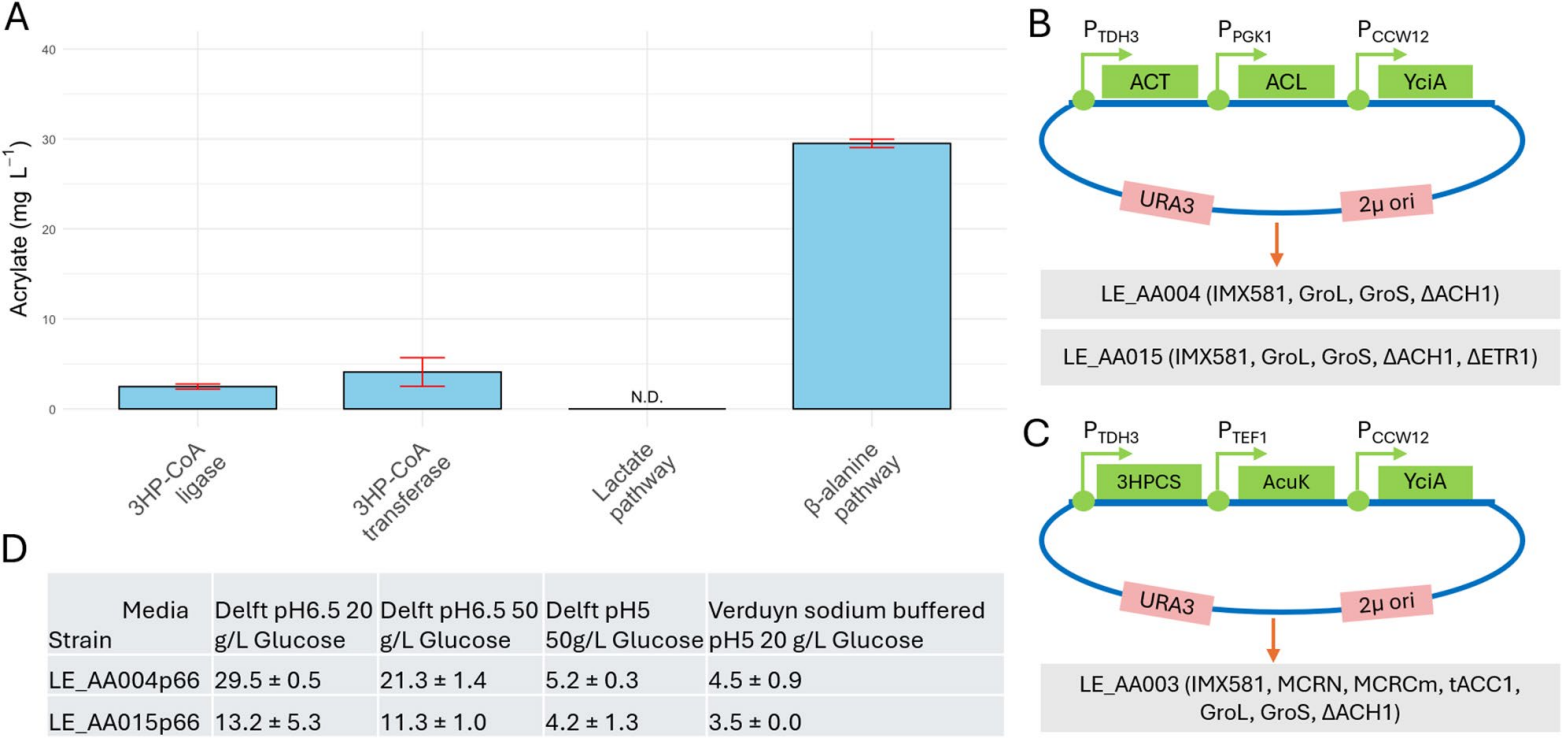
Overview of the results from the pathway screening. **A** Maximal obtained AA titre from the best-performing enzyme combination for each pathway. Bar plots represent the mean of *n* = 3 biological replicates, error bars indicate standard deviation. N.D. = Not detected. **B** Plasmid design and respective background strains used for the β-alanine pathway. Strain LE_AA015 was only used for optimisation of production. **C** Plasmid design and background for the 3HP-CoA ligase pathway. The setups for the 3HP-CoA transferase and the lactate pathway followed the same plasmid scheme. A summary of strain genotypes is provided in panels B and C, where only pathway-relevant genetic modifications are listed. All strains were based on *S. cerevisiae* IMX581 and additionally expressed the *E. coli* chaperons *GroL* and *GroS*. A complete overview of strain modifications and constructs is available in Supplementary Table 3. **D** Acrylate titres obtained under different media conditions for the β-alanine pathway in two background strains

To assess the performance of the different pathways (Fig. 1), we initially focused on enzymes that had been previously shown to work in *E. coli* for the β-alanine [10] and the 3HP pathway [8] and were hence chosen as a starting point for evaluation in *S. cerevisiae*. A schematic overview of the screening design, including plasmid architecture, promoter usage, and strain background, is shown in Fig. 4B and C. Early experiments indicated two things: [1] the β-alanine route outperformed the 3HP route under the tested conditions (Supplementary Fig. 4) and [2] the rate limiting step appeared to be the AA release, as the addition of an acryloyl-CoA reductase resulted in a two-order-of-magnitude increase in propionic acid titres using the 3HP route (Supplementary Fig. 5).

These initial observations and enzyme combinations guided subsequent pathway optimisation for screening the different catalytic steps. Notably, these experiments were performed in an ACH1-positive background prior to identification of the degradation target.

### Screening of the β-alanine pathway

Based on the prescreening results, the β-alanine pathway was selected for further evaluation of candidate enzymes involved in the AA releasing step. During prescreening, co-expression of the *E. coli* chaperons GroL and GroS improved AA titres and was therefore included in all subsequent experiments (Supplementary Fig. 6). In contrast, supplementation of β-alanine at concentrations from 0.5 to 3 g L^− 1^ did not have a significant effect on AA titres (Supplementary Fig. 7). Hence, 3 g L^− 1^ was used in all conditions to ensure β-alanine availability when screening this pathway.

Most of the enzymes screened for the AA release step showed a final titre of ~28 mg L^− 1^ (Supplementary Fig. 8 A). Normalising AA production to OD_600_, identified two targets with higher performance (Supplementary Fig. 8B). We determined their respective product formation rate and figured that the *yciA* from *E. coli* exhibited the highest AA formation rate (Supplementary Fig. 9). We therefore concluded that *Ec_yciA* is the least rate limiting enzyme of the ones tested.

Next, we screened six β-alanyl-CoA ammonia-lyases of which four showed detectable activity with *acl2* from *Anaerotignum propionicum* (*Clostridium propionicum)* having the highest titre (Supplementary Fig. 10). Lastly, a test of enzymes for transferring CoA onto β-alanine showed that only *Cp_act* supported significant activity. Neither the CoA transferase from *C. necator* (*Cn_pct*) nor the propionyl-CoA transferase from *A. propionicum* (*Cp_pct*) showed more than marginal activity resulting in titres of around 1 mg L^− 1^ (Supplementary Fig. 11), which for the latter is consistent with previous results [33]. Therefore, within the β-alanine pathway, the best performing enzyme combination screened was the combination of *Cp_act*, *Cp_acl2* and *Ec_yciA*, resulting in a maximum titre of 30 mg L^− 1^.

### Screening of the 3HP pathways

Based on the previous results, *Ec_yciA* was also used for the last step of the pathway to release AA. The background strain (LE_AA3) carried in addition to the *E. coli* chaperons also the 3 HP biosynthetic genes from *Chloroflexus aurantiacus* (*MCRN*, *MCRCm*) as previously described [46–48]. Out of 8 enzymes tested for their activity to catalyse the 3HP-CoA dehydration to acryloyl-CoA, multiple strains yielded a similar titre of ~2.1 mg L^− 1^ (Supplementary Fig. 12 A). Normalization to OD_600_ also showed no significant differences (Supplementary Fig. 12B) and we continued with the 3-hydroxybutyryl-CoA dehydratase from *Clostridium acetobutylicum* (*Ca_crt*) for further experiments.

Next, we tested enzymes catalysing the transferring of a CoA group onto 3HP. Out of the tested enzymes for this step, only the 3-hydroxypropionyl-CoA synthetase from *Sulfurisphaera tokodaii* (*St_stk_07830*), which was also used for the respective step in the previous screening rounds, generated a titre above 1 mg L^− 1^ (Supplementary Fig. 13).

In parallel, we also tested a *Halomonas* CoA-transferase (*Hs_acuN*) [21], which links the activation of 3HP to AA release (Fig. 1B). Using the 3HP-CoA dehydratase from the same species (*Hs_acuK*) this combination yielded more than 4 mg L^− 1^, outperforming all other 3HP pathway designs (Supplementary Fig. 12 A). Despite these optimizations, the 3HP route remained less efficient than the β-alanine pathway. Although self-production of 3HP may limit flux, > 1 g L^−1^of 3HP was detected at the end of cultivation (Supplementary Fig. 14), suggesting precursor supply alone does not explain the low performance of the pathway. To confirm this hypothesis, we tested the respective enzyme combinations in the non-3HP producing background strain used for screening of the β-alanine pathway (LE_AA4) and supplemented 3 g L^− 1^ of 3HP at the beginning of cultivation. Nevertheless, the resulting AA titres did not exceed 5 mg L^− 1^ (Supplementary Fig. 15).

### Testing the lactate pathway

Testing of the lactate pathway was performed under anaerobic conditions due to the lactoyl-CoA dehydratase (LCD) enzyme complex being inactivated by oxygen [22]. We integrated the lactate dehydrogenase gene from *Lactococcus cremoris* (*Lc_ldh*) into the genome but kept the other pathway genes on a plasmid-based system like before. Expressing *Lc_ldh* in strain LE_AA013p78 resulted in lactate formation in the g L^−1^ range, confirming functional lactate formation. However, despite functional lactate production, the strain harbouring the entire pathway (LE_AA013p78) exhibited very slow growth with a long lag phase and produced no detectable AA (data not shown). This could be due to several reasons. First, the enzyme complex LCD responsible for lactyl-CoA dehydration, consisting of two subunits (EIIα and EIIβ) and an activator (EI), might have not assembled correctly leading to a nonfunctional complex or the traces of oxygen in our setup might have been enough for inactivation. Additionally, the complex also requires [Fe-S] clusters [49, 50] which are normally not abundant in *S. cerevisiae* and have required upregulation of the [Fe–S] cluster metabolism for other [Fe-S] dependent dehydratases to work [51, 52]. Further, the low equilibrium ratio of both acrylate/lactate or their respective CoA esters with around 3% and 0.5% respectively [23, 53], might hinder conversion.

### Continued acrylic acid degradation during pathway expression

During pathway screening, we observed that engineered strains harbouring the pathway genes again degraded self-produced AA. To identify the responsible gene, we tested 4 β-alanine pathway variants that showed activity in the previous screening (Supplementary Fig. 16). All 4 strains quickly degraded externally added AA, and *Cp_act* was the only common gene in the settings. The control strain, containing an empty plasmid, also degraded AA to some extent under these conditions (Supplementary Fig. 16) and displayed a normal ethanol growth phase despite *ΔACH1*, in contrast to earlier experiments performed in YPD where no AA degradation was observed. Ethanol growth capabilities of *ΔACH1* strains has been shown to be pH related in earlier reports [28, 31]. We then discovered degradation mainly occurred after glucose depletion, suggesting a link to respiratory metabolism.

Retesting at pH 5 confirmed this: the control no longer showed respiratory growth, nor degraded externally added AA, while the strains carrying the pathway genes exhibited severe growth impairment with a long lag phase yet also almost no AA degradation (Supplementary Fig. 17). These results indicate that AA degradation is indeed linked to respiratory activity, which in turn is pH dependent. Given that three key ALE targets (*ACH1*, *YBR238C* and *ETR1*) are mitochondrial, we examined *ETR1* more closely due to its link to respiration [34]. The double mutant *ΔACH1 ETR1*^M1V^ showed the highest AA tolerance, whereas *YBR238C* lacked similar annotation nor showed the double mutant further tolerance improvement.

We therefore tested AA degradation again in *ΔACH1 ETR1*^M1V^ and *ΔACH1* Δ*ETR1* mutants with the control plasmid or the pathway genes. The *ΔACH1 ETR1*^M1V^ strain behaved very similarly to the *ΔACH1*, partially degrading AA (control plasmid) or fully (pathway expression). The *ΔACH1* Δ*ETR1* strain however, no longer showed respiratory growth which is in agreement with the previous work on this gene [34], nor did it degrade exogenous AA at all (control plasmid) or just marginally (pathway plasmid) (Supplementary Fig. 18). These results support a direct role of ETR1p in AA degradation.

### Testing alternative conditions for acrylic acid production

Finally, we wanted to see whether cultivation conditions could be tweaked based on our previous results to obtain higher AA titres. Therefore, we decided to test the producer strains of the β-alanine pathway with both *ΔACH1* or *ΔACH1 ΔETR1* background (Fig. 4B) in Delft pH 6.5 with 50 g L^− 1^ of glucose to potentially prolong the production time before respiratory degradation by the *ΔACH1* producer strain. As lower pH also inhibited degradation, we also tested Delft pH 5 with 50 g L^− 1^ glucose and lastly also tested our original evolution media buffered at pH 5 to see if production could be improved in either condition.

However, no increase of AA titre was observed under either condition (Fig. 4D). The low production at pH 5 likely resulted from the growth impairment of the *ΔACH1* based strain. Increasing the glucose concentration to 50 g L⁻¹ lowered the pH of the media at the end of fermentation (from 5.7 to 4.7 compared with 20 g L⁻¹glucose, Supplementary Fig. 19) leading to similar growth limitations even when starting at pH 6.5. The *ΔACH1 ΔETR1* producer strain showed slower growth, lower biomass yield across all conditions and incomplete ethanol consumption, likely limiting AA production. When normalised to biomass, the resulting titres for Delft pH 6.5 with 20 or 50 g L⁻¹glucose, were comparable to but overall lower than those of the *ΔACH1* based strain (Supplementary Fig. 20).

## Conclusion

In this study we applied ALE to obtain *S. cerevisiae* strains with increased tolerance to acrylic acid and identified the underlying genetic causes for tolerance phenotypes. Our results showed that tolerance to AA seems to be directly linked to mitochondrial function. Deletion of the mitochondrial CoA transferase *ACH1* significantly increased tolerance to AA and the removal of the mitochondrial signal peptide from the 2-enoyl thioester reductase *ETR1* further improved the tolerance phenotype. Additionally, we found *YBR238C* as a target whose single deletion could improve AA tolerance although no additive effect was observed in combination with the *ACH1* deletion. In addition to tolerance against acrylic acid, we could also show that *ACH1* is mainly responsible for AA degradation.

Beyond tolerance engineering we further evaluated different biosynthetic pathways for their potential in direct fermentative production of AA and identified the β-alanine pathway as the most promising. Using this route, we produced AA in yeast up to 30 mg L^− 1^ (Fig. 4A).

## Methods

### Plasmids and strains

All plasmids used in this study can be found in Supplementary Table 2. Propagation and cloning were performed in *E. coli* strain DH5α. An overview of the *S. cerevisiae* strains used is available in Supplementary Table 3. All exogenous genes incorporated into the yeast were synthesised by Twist Bioscience or IDT and codon-optimized for efficient expression in *S. cerevisiae* (Supplementary Table 4). Unless stated otherwise, strains were constructed in the CRISPR-ready *S. cerevisiae* strain IMX581 [54].

### Media

*E. coli* strains were selected and cultivated on Luria-Bertani (LB) agar plates, supplemented with the respective antibiotic for selection (100 mg L^−1^ Ampicillin, 50 mg L^− 1^ Kanamycin, 30 mg L^− 1^ Chloramphenicol). Liquid cultures were grown in LB medium with the same respective antibiotic concentration. Yeast cells were grown in YPD medium containing 10 g L^− 1^ yeast extract, 20 g L^− 1^ peptone and 20 g L^− 1^ glucose. For strains containing a URA3-based plasmid, selection was done using synthetic complete media without uracil (SD-Ura) comprised of 6.9 g L^− 1^ yeast nitrogen base without amino acids (YNB), 0.77g L^− 1^ complete supplement mixture without uracil (CSM-ura), 20 g L^− 1^ glucose and 20 g L^− 1^ agar powder. Counterselection against the *URA3* marker was done using SD +5’-FOA plates, containing 6.7 g L^− 1^ YNB, 0.78 g L^− 1^ CSM, and 1 g L^− 1^ 5-fluoroorotic acid (5-FOA). Yeast strains containing a kanMX resistance gene were selected on YPD plates supplemented with 200 mg L^− 1^ G418. Most cultivation was performed in Delft shake flask medium containing (L^− 1^) 7.5 g (NH4)_2_SO_4_, 14.4 g KH_2_PO_4_, 0.5 g MgSO_4_•7H_2_O, 2 mL trace metal solution and 1 mL vitamin solution with pH set to 6.5 using KOH if not otherwise stated. Glucose (20 g L^− 1^) was used as carbon source if not otherwise stated. β-alanine, 3HP or AA was added to the medium when required for the specific experiment. β-alanine was supplemented for pathway screening of the β-alanine route, 3HP was added during one screening experiment, while AA was added for evolution experiments or degradation assays as indicated. Vitamin and trace metal solution were prepared as previously described [55]. For the evolution and evolved clone testing, we used Verduyn media [55] buffered at pH 5 with 97 mL L^− 1^ 0.5 M Citric acid and either 103 mL L^− 1^ Na_2_HPO_4_ or K_2_HPO_4_, as previously used for organic acid tolerance evolution [29, 40]. Uracil was supplemented to the media at a concentration of 60 mg L^− 1^ where applicable. For anaerobic cultivation, ergosterol and Tween80 were added at concentrations of 10 mg L^− 1^ and 420 mg L^− 1^ respectively.

### Plasmid and strain construction

Plasmids were constructed using the MoClo toolkit and its expansion when applicable [56, 57]. For plasmids to be transformed into *S. cerevisiae*, we first built a MoClo level 2 plasmid containing *URA3* as yeast selection marker, a 2µ origin, a bacterial kanamycin resistance and a bacterial GFP dropout for selection (pLE_YTK007). The respective pathway genes were then added in order in the GFP dropout site. Guide RNA (gRNA) sequences were identified using the benchling crispr tool. Plasmid correctness was confirmed by Sanger or Whole Plasmid sequencing performed by Eurofins Genomics. We employed CRISPR/Cas9-based techniques for gene integration, deletion and mutation similarly as previously described [54]. Transformation of plasmids and fragments into *S. cerevisiae* was done following a modified version of the lithium acetate method [58] using 10% (v/v) DMSO prior to heat shock with subsequent selection on SD-ura plates. The genomic modification was confirmed by diagnostic PCR. Deletions were confirmed based on the expected change in amplicon size, while point mutations were validated by PCR amplification of approximately 500 bp surrounding the targeted locus and Sanger sequencing of the amplicon.

### Growth screening

Growth tests were conducted in a Growth profiler 960 (Enzyscreen) using 96-half-deepwell microplates with a culture volume of 250 µL. Individual colonies were inoculated into 5 mL of the corresponding medium used for the subsequent growth experiment and incubated at 30 °C with 200 rpm agitation. The precultures were used to inoculate the plates to a starting OD_600_ of 0.1 and incubated at 30 °C and 250 rpm agitation. Maximum specific growth rates for the strains were calculated using the maximum slope in plots of ln (OD) versus time over a period of at least 2.5 h.

### Adaptive laboratory evolution

For the ALE the strain GL01 [29] was used as the original parental strain. A single colony of the strain was inoculated into Verduyn sodium buffered medium and grown overnight. The grown cells were used to inoculate five independent 100 mL shake flasks, each containing 20 mL of the same medium supplemented with 400 mg L^− 1^ AA as the starting concentration (see Results), to an initial optical density at 600 nm (OD) of 0.1. Cultures were grown for 24 h in an Aquila bioshaker with live lightscatter monitoring at 30 °C and 200 rpm. After 24 h, cultures were reinoculated to an OD of 0.1. The citrate–phosphate buffered medium maintained a stable pH of 5 throughout the evolution experiment, which was verified by pH measurements at the end of selected cultivation cycles. AA concentration was gradually increased by steps of 50 or 100 mg L^− 1^ when the respective culture finished glucose phase within the 24 h incubation period. Number of generations through the evolution was calculated by summing the logarithmic difference of starting and end OD of each transfer. Cells were cultured for ~50 serial transfers, corresponding to approximately 215 to 231 generations. Culture purity was routinely monitored by fluorescence microscopy based on the GFP marker present in GL01. Samples of the evolving populations were periodically collected every five days and stored at −80 °C. Single clones were isolated by plating 50 µL of the culture on YPD G418 agar, and up to 6 clones per population were randomly picked for further characterisation.

### DNA extraction and sequencing and analysis

Single isolates were grown overnight in YPD. The Yeast DNA Extraction Kit (ThermoFisher Scientific) was used to extract genomic DNA using the respective protocol. Sequencing was performed by Eurofins Genomics using the INVIEW Resequencing service on an Illumina NovaSeq X platform with paired-end 2 × 150 bp reads, achieving an average coverage depth of ~140x. Analysis of the obtained Illumina reads was done using Breseq v0.34.1 [59] with bowtie2 version 2.4.4 [60] as the aligner to map the reads against the annotated genome of GL01 [29]. Breseq was run in polymorphism mode with polymorphism-frequency-cutoff set to 0.2 and the option junction-alignment-pair-limit set to 0 (no limit). False positives were filtered out by comparing the called mutations for the evolved clones versus the ones called for the starting strain. Putative chromosomal duplications were identified based on consistent increases in read coverage across genomic regions in Breseq-generated coverage plots.

### Enzyme selection for pathway screening

Candidate enzymes for pathway screening were identified based on two criteria: [1] well-documented catalytic activity for the target reaction in the published literature, and [2] reported kinetic parameters in the BRENDA database. For the latter, enzymes were selected based on comparatively favourable kinetic properties e.g., lower Km or higher k_cat_ values, relative to other reported enzymes catalysing the same or closely related reactions. A complete set of all heterologous enzymes chosen for screening is listed in Supplementary Table 4.

### Pathway screening

Single colonies of the strains selected for β-alanine and 3HP pathway screening (see Supplementary Table 3) were picked from SD-URA plates, inoculated into 5 mL Delft medium and grown 16–48 h at 30 °C with 200 rpm agitation. Main cultivation was performed in 100 mL shake flasks containing 20 mL of the respective medium tested (see Results), inoculated to a starting OD of 0.1 and incubated under the same conditions. Samples for metabolite analysis were collected at regular intervals depending on the experiment. Typically, every 12–24 h during initial screening, every 4 h during rate measurements, and approximately every 24 h during anaerobic cultivation once growth was observed. For each sampling point 1 mL of culture was taken, centrifuged at 14 000 g and 800 µL of the supernatant was collected and stored at −20 °C until analysis. For testing of the lactate pathway, colony picking and precultures were handled the same. Main cultivation was performed in 50 mL Falcon tubes using anaerobically prepared medium, incubated at 30 °C with 220 rpm agitation, and cultivated in an anaerobic chamber keeping oxygen concentrations near 1 and at least below 20 ppm.

For optimisation experiments, producer strains were cultivated in Delft medium at pH 5 or 6.5 with 50 g L⁻¹ glucose, or in the evolution medium, as described in the Results section (Fig. 4D).

### Metabolites analysis

1 mL of cultured sample was taken and the cells separated from the supernatant by centrifuging at 14 000 g for 2 min. Metabolite analysis was performed using a Shimadzu-VWD HPLC system equipped with a Aminex HPX-87 H column. The column was maintained at 40 °C, 5 mM H_2_SO_4_ served as mobile phase with a flow rate of 0.45 mL/min.

## Supplementary Information

Supplementary Material 1. Supplementary Fig: Supplementary Figures, Contains all the supplementary figures referenced in the text.

Supplementary Material 2. Supplementary Table 1: Mutations, Contains information about all mutations found during the whole genome resequencing.

Supplementary Material 3. Supplementary Table 2: Plasmids, Contains information about all plasmids used in this study.

Supplementary Material 4. Supplementary Table 3: Strains, Contains information about all strains used in this study.

Supplementary Material 5. Supplementary Table 4: Genes, Contains the sequence of all heterologous genes used in the pathway screening.

## Data Availability

The datasets used and/or analysed during the current study are available from the corresponding author on reasonable request.
