# Supplementary material for: Establishing Saccharomyces cerevisiae as a host for renewable acrylic acid production
Source: Microb Cell Fact. 2026 Mar 4;25:93. doi: 10.1186/s12934-026-02974-3 (PMC13067684; doi:10.1186/s12934-026-02974-3)
Supplement: Supplementary file 5 — Supplementary Material 5. Supplementary Table 4: Genes, Contains the sequence of all heterologous genes used in the pathway screening. [file 12934_2026_2974_MOESM5_ESM.docx]

Establishing Saccharomyces cerevisiae as a host for renewable acrylic acid production

Leon Eisentraut^1^, Xiaowei Li^1, 2^ and Yun Chen^1, 3^

^1^Department of Life Sciences, Chalmers University of Technology, Gothenburg, Sweden

^2^Tianjin Institute of Industrial Biotechnology, Chinese Academy of Sciences, Tianjin, China

^3^Novo Nordisk Foundation Center for Biosustainability, Technical University of Denmark, Kongens Lyngby, Denmark

Supplementary File


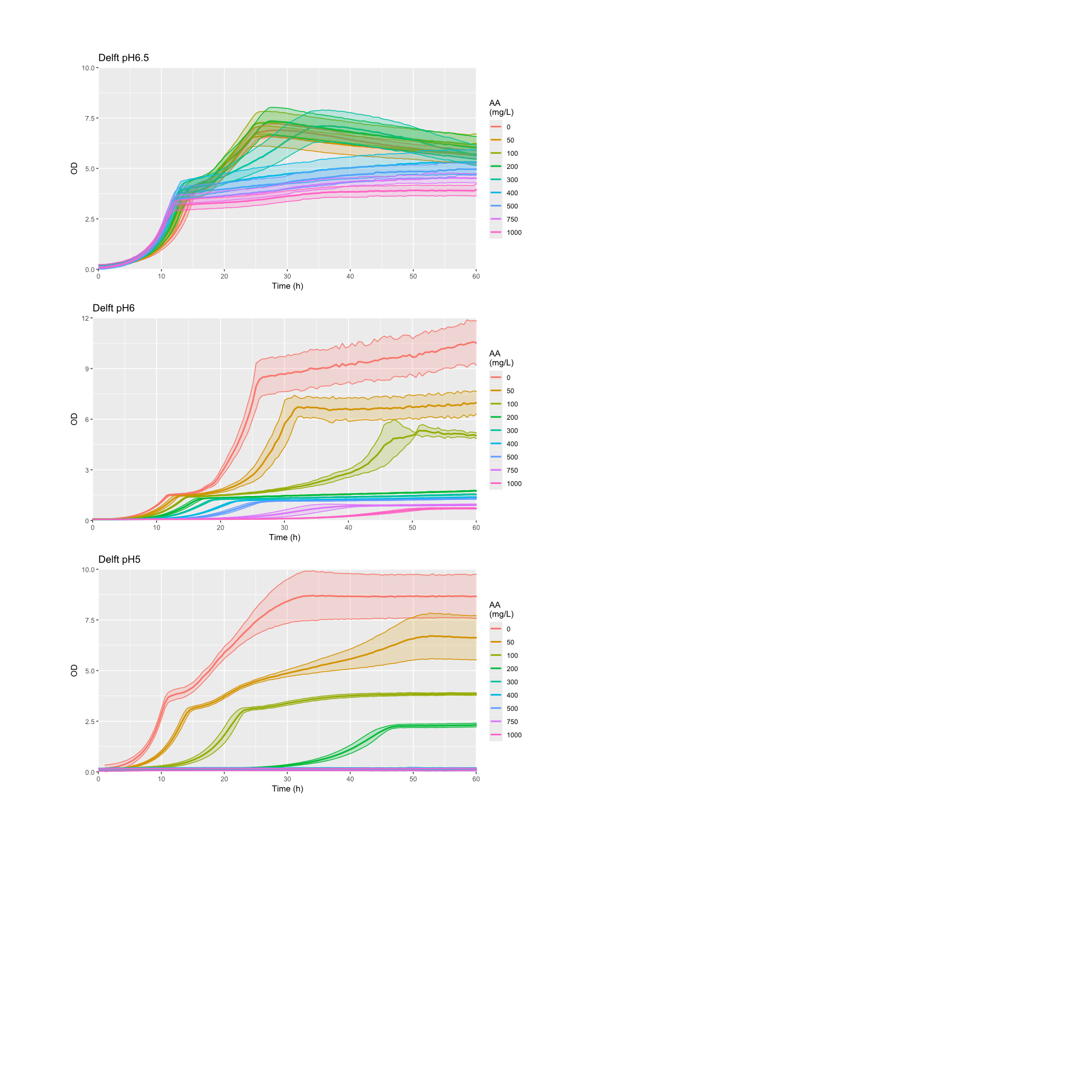


Supplementary Figure 1. Effect of AA on the growth *S. cerevisiae* CEN.PK113-7D at different pH. The graphs show the mean (thick line) and standard deviation (shaded area) of n=5 (pH6.5 and pH5) and n=3 (pH6) biological replicates.


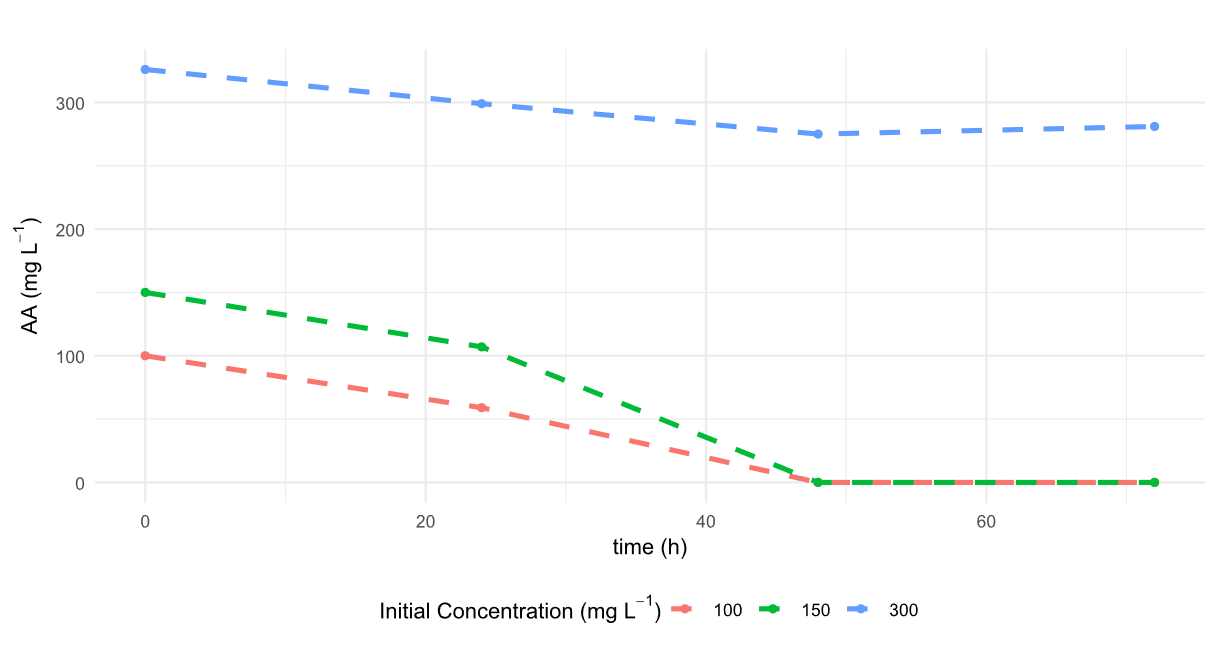


Supplementary Figure 2. Degradation of supplemented AA by *S. cerevisiae* CEN.PK113-7D over time in Delft pH 6.5 during shake flask cultivation.


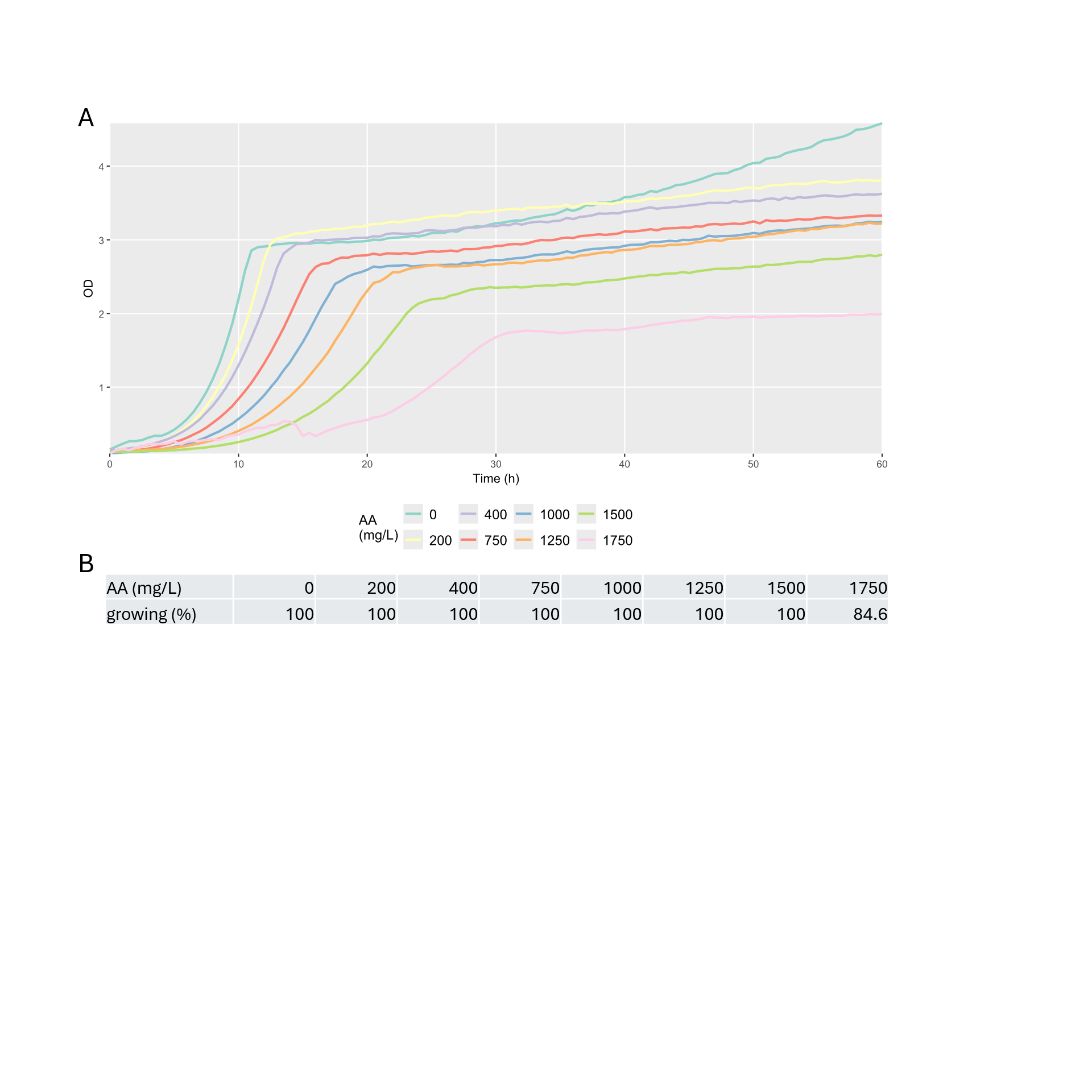


Supplementary Figure 3. Growth curve of isolate ALE2_1 under different AA concentrations in the evolution media (A). Percentage of isolates growing at a maximum specific growth rate above 0.05 at the respective AA concentration (B).


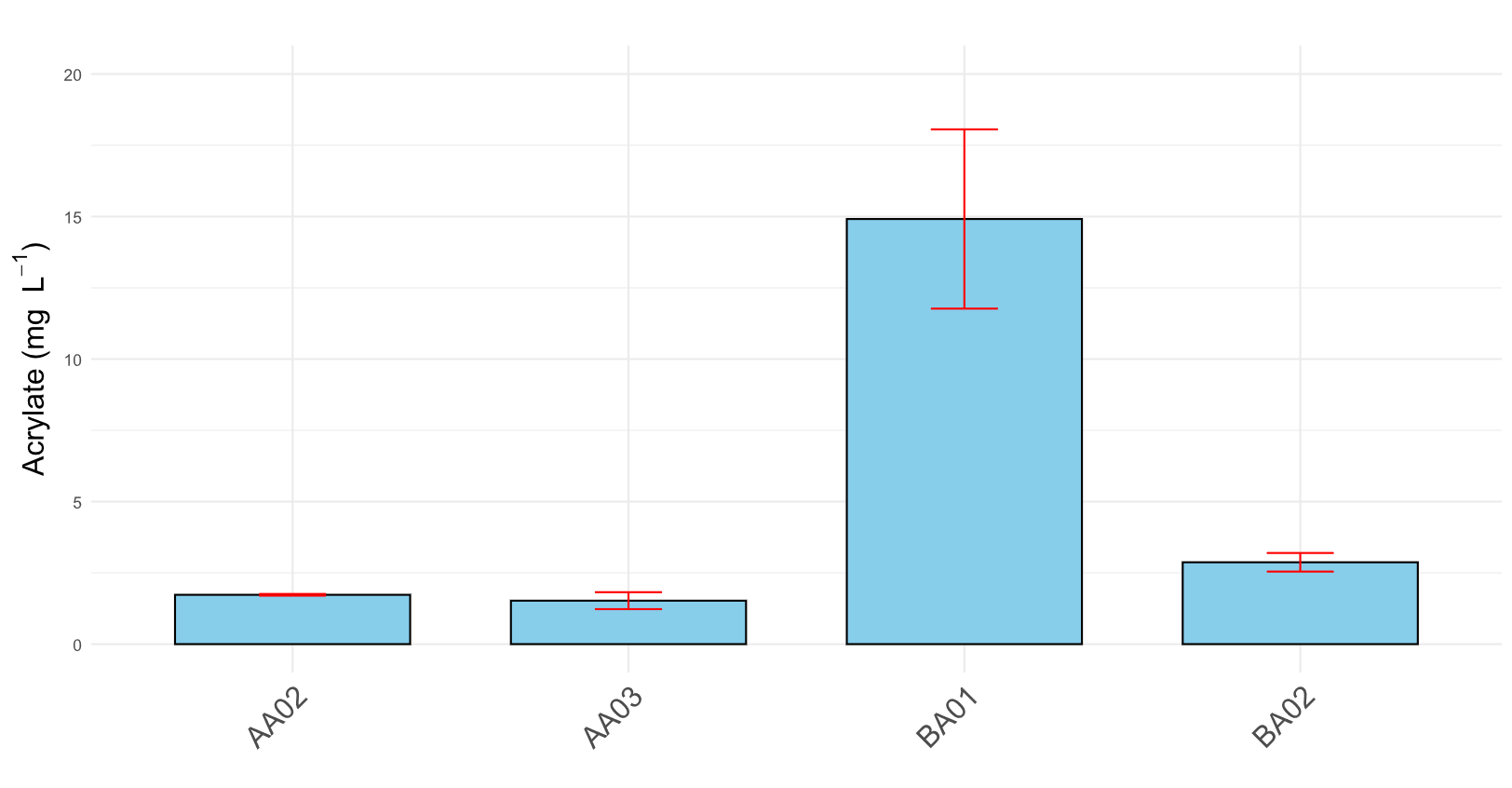


Supplementary Figure 4. Comparison of the 3HP pathway (AA02 and AA03) and the β-alanine pathway (BA01 and BA02) during the prestudy. Bar plots represent the mean of n=3 biological replicates, error bars indicate standard deviation. Enzyme combinations for each strain are listed in Supplementary Table 3.


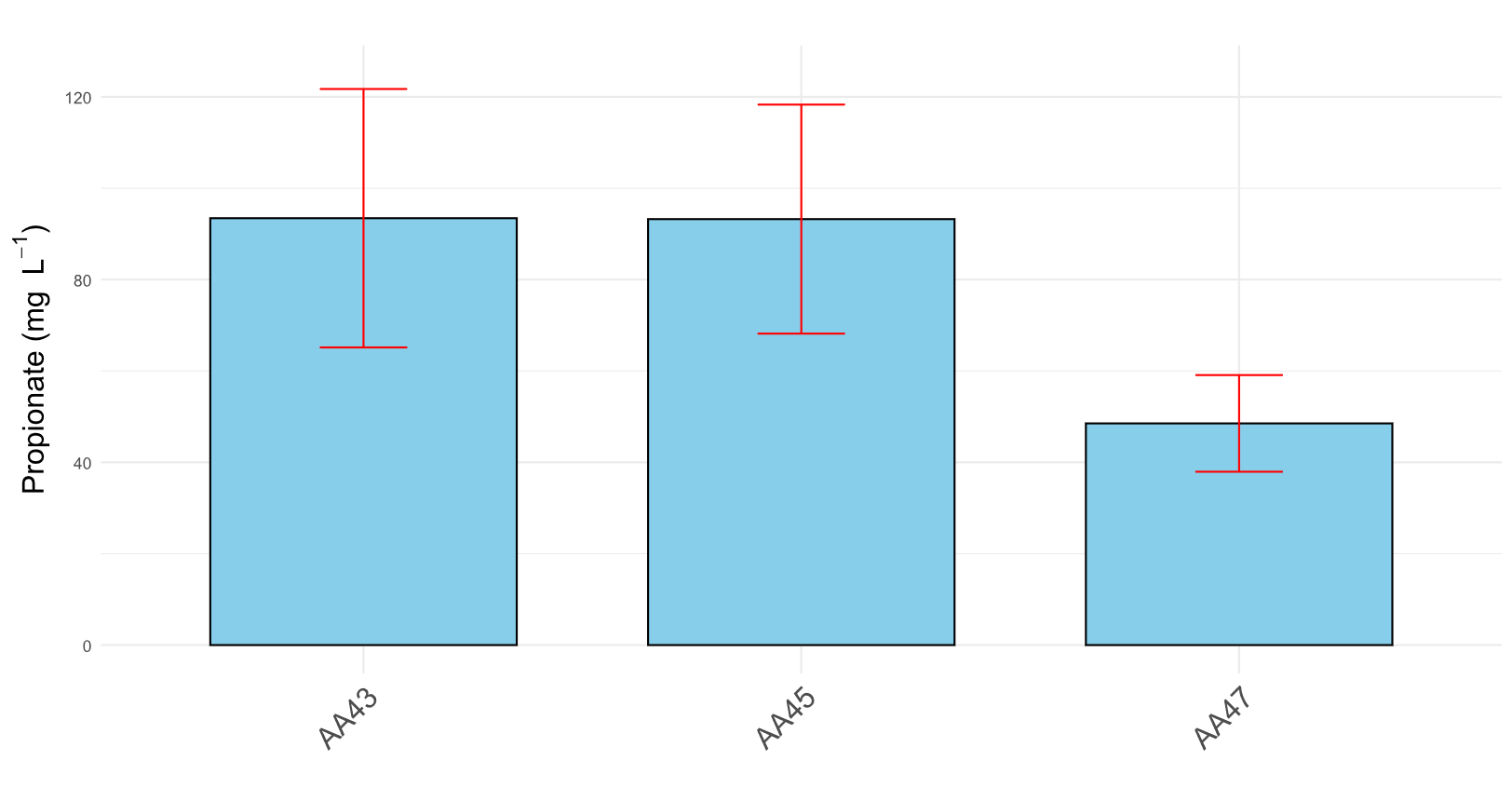


Supplementary Figure 5. Propionate titres measured after expression of different acryloyl-CoA reductases using the 3HP route during the prestudy. Bar plots represent the mean of n=3 biological replicates, error bars indicate standard deviation. Enzyme combinations for each strain are listed in Supplementary Table 3.


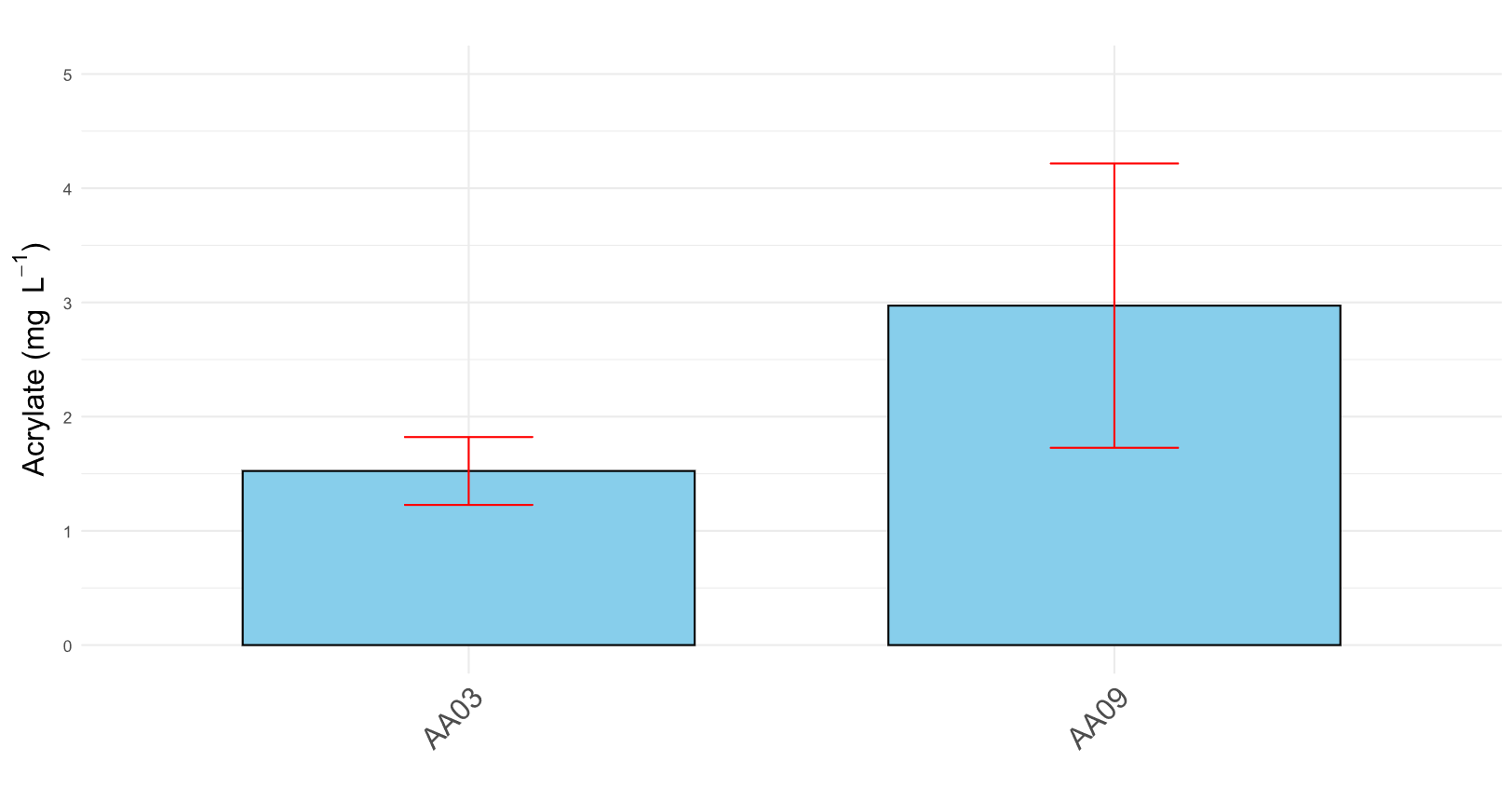


Supplementary Figure 6. Effect of *E. coli* chaperones GroL and GroS on AA titres. Comparison between strain expressing the chaperons (AA09) and a strain without (AA03). Enzyme combinations for each strain are listed in Supplementary Table 3. Bar plots represent the mean of n=3 biological replicates, error bars indicate standard deviation.









BA03

BA02

BA01

Supplementary Figure 7: Effect of β-alanine supplementation at different concentrations on AA titres in different strains. Enzyme combinations for each strain are listed in Supplementary Table 3. Data represent single measurements from individual experiments.


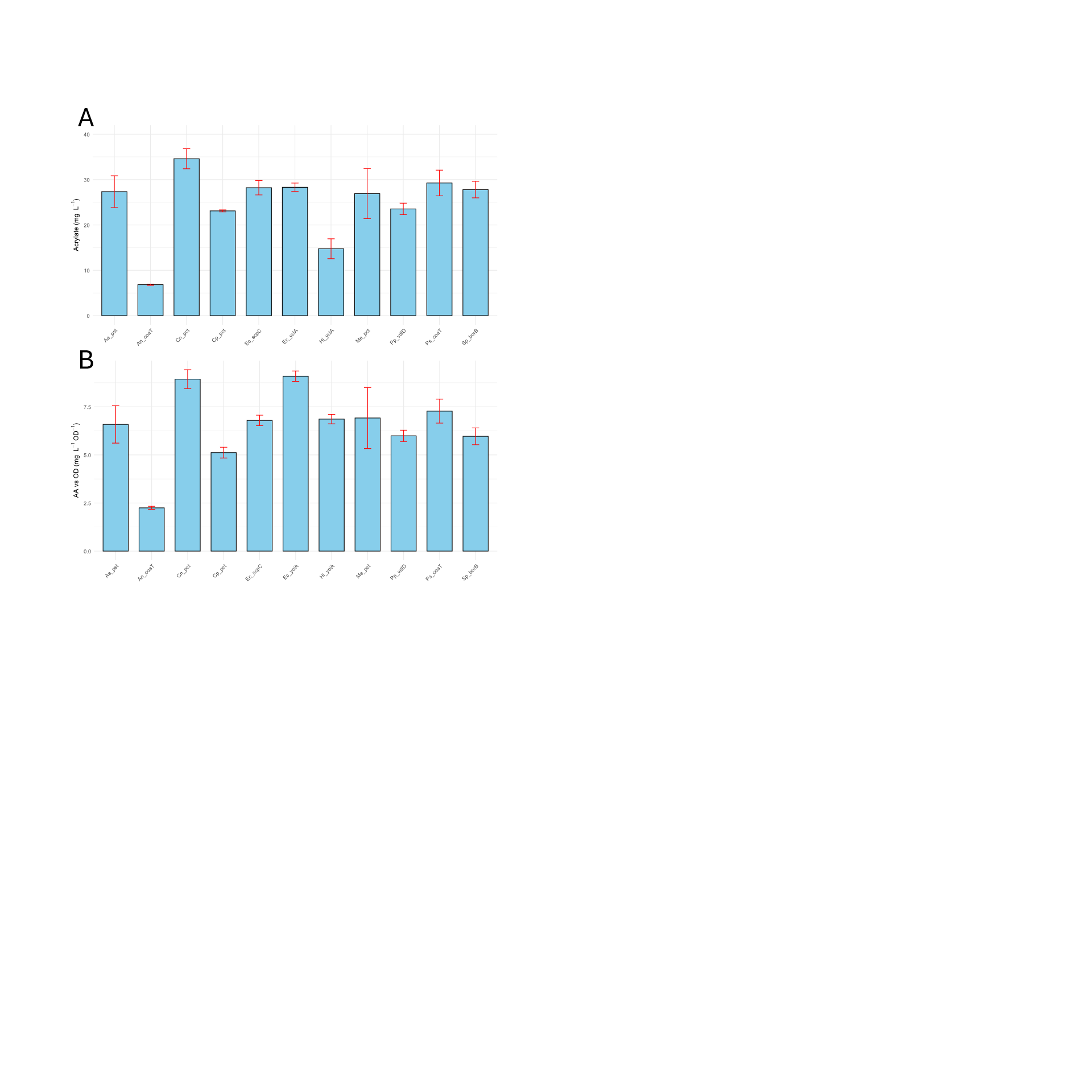


Supplementary Figure 8. Screening of AA-releasing enzyme in the β-alanine pathway. (A) measured AA titres of the tested strains. (B) AA titre normalised to biomass (OD_600_) for the same strains. Bar plots represent the mean of n=3 biological replicates, error bars indicate standard deviation.


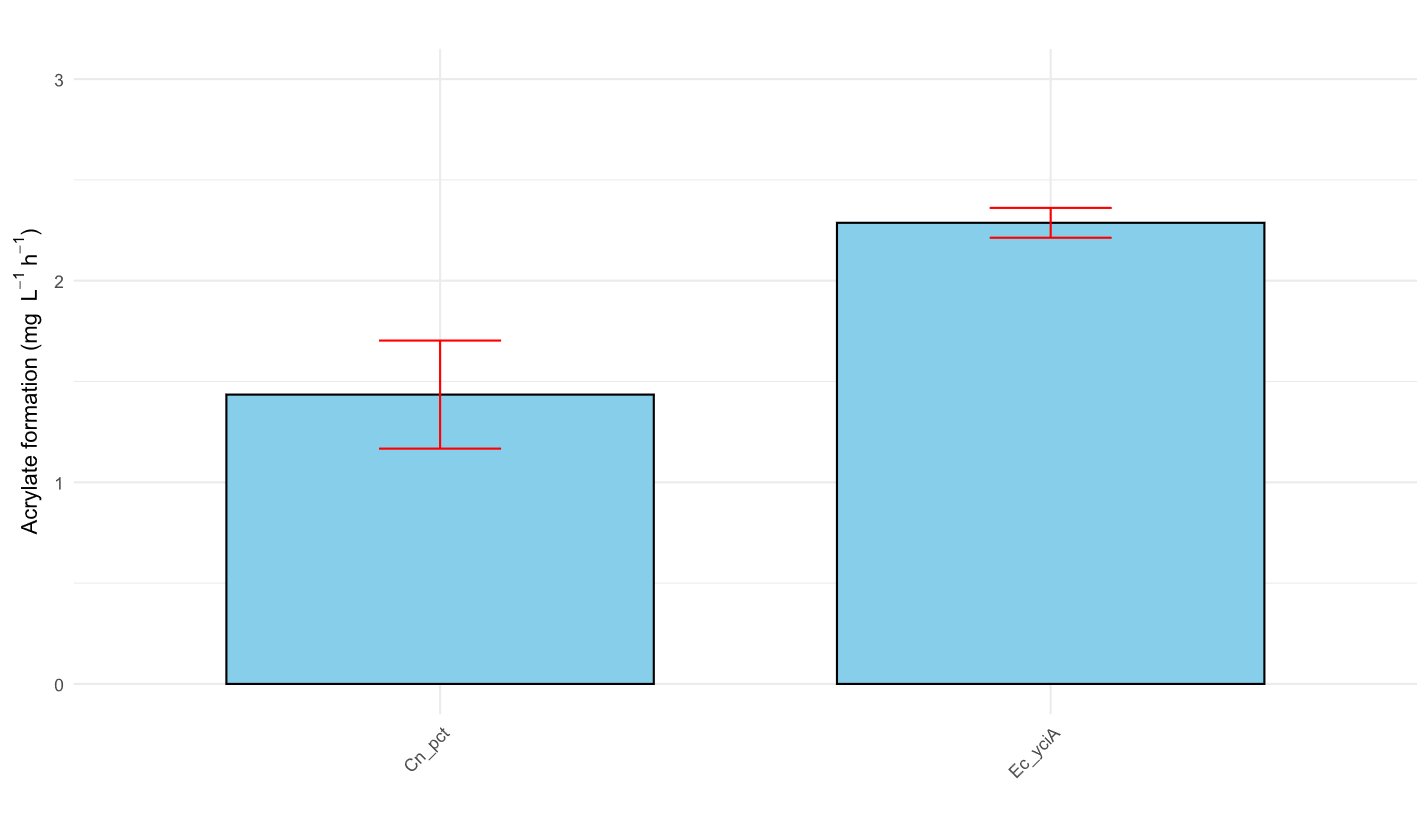


Supplementary Figure 9. Rates determined for acrylate formation in the tested strains. Bar plots represent the mean of n=3 biological replicates, error bars indicate standard deviation.


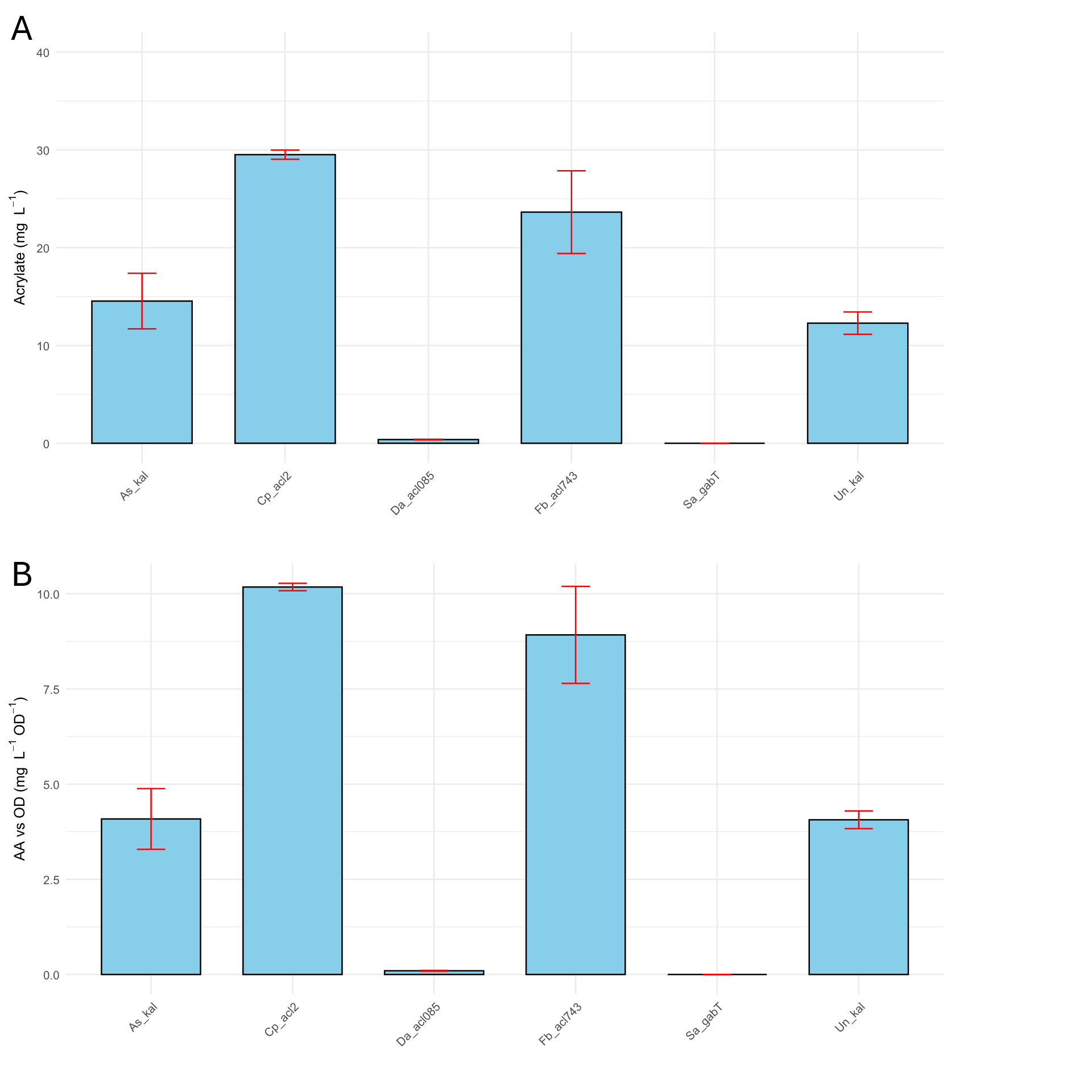


Supplementary Figure 10. Screening of enzymes catalysing the β-alanyl-CoA ammonia-lyase reaction. Bar plots represent the mean of n=3 biological replicates, error bars indicate standard deviation.


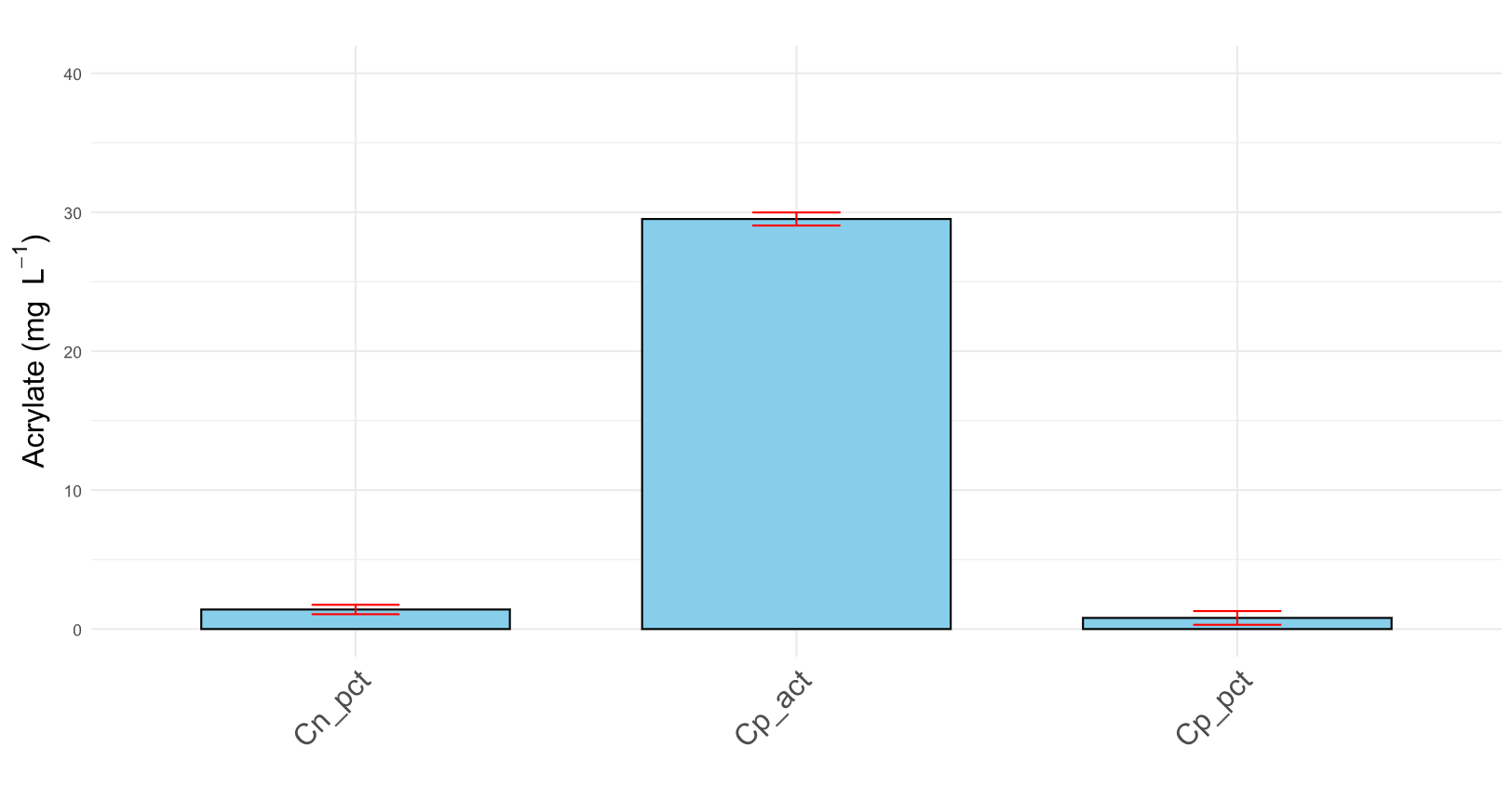


Supplementary Figure 11. Screening of enzymes catalysing the transfer of a CoA moiety onto β-alanine. Bar plots represent the mean of n=3 biological replicates, error bars indicate standard deviation.


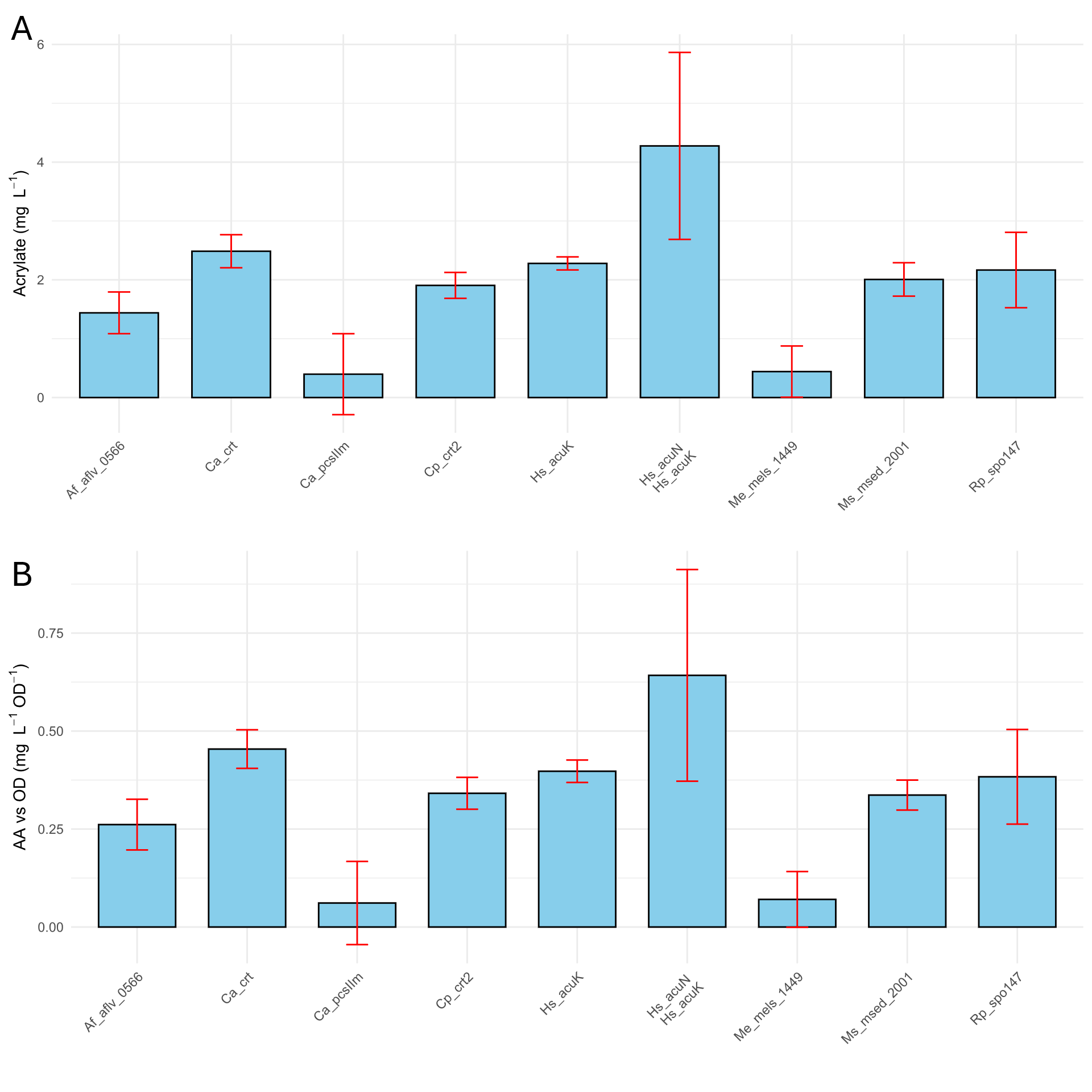


Supplementary Figure 12. Screening of enzymes dehydrating 3HP-CoA to AA-CoA. The *Hs_acuN*/*Hs_acuK* double gene system employs a 3HP-CoA:acrylate CoA-transferase in contrast to the 3HP-CoA ligase used in the other tested systems. (A) measured AA titres of the tested strains. (B) AA titre normalised to biomass (OD_600_) for each strain. Bar plots represent the mean of n=3 biological replicates, error bars indicate standard deviation.


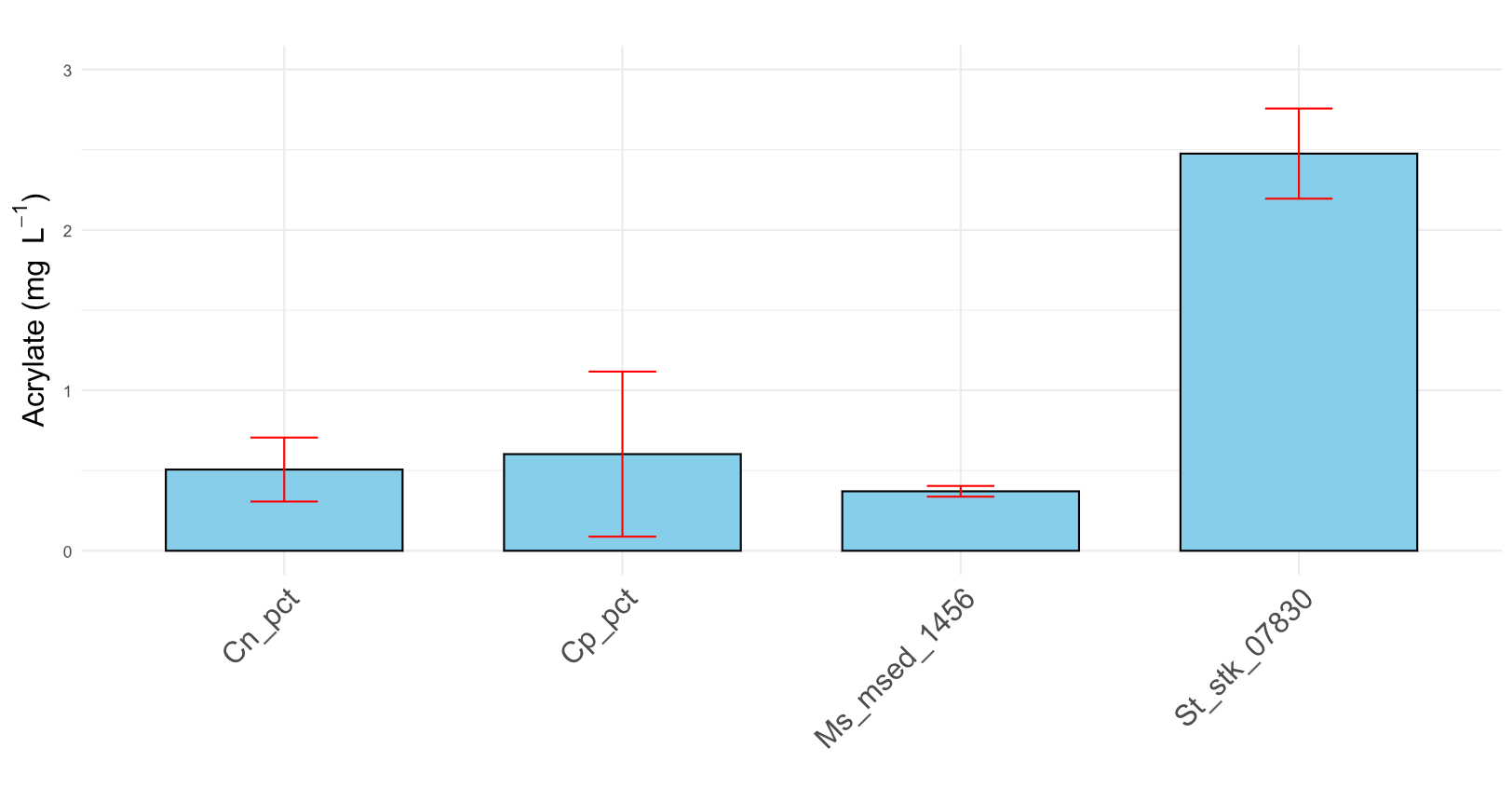


Supplementary Figure 13. Screening of enzymes catalysing the transfer of a CoA moiety onto 3HP. Bar plots represent the mean of n=3 biological replicates, error bars indicate standard deviation.


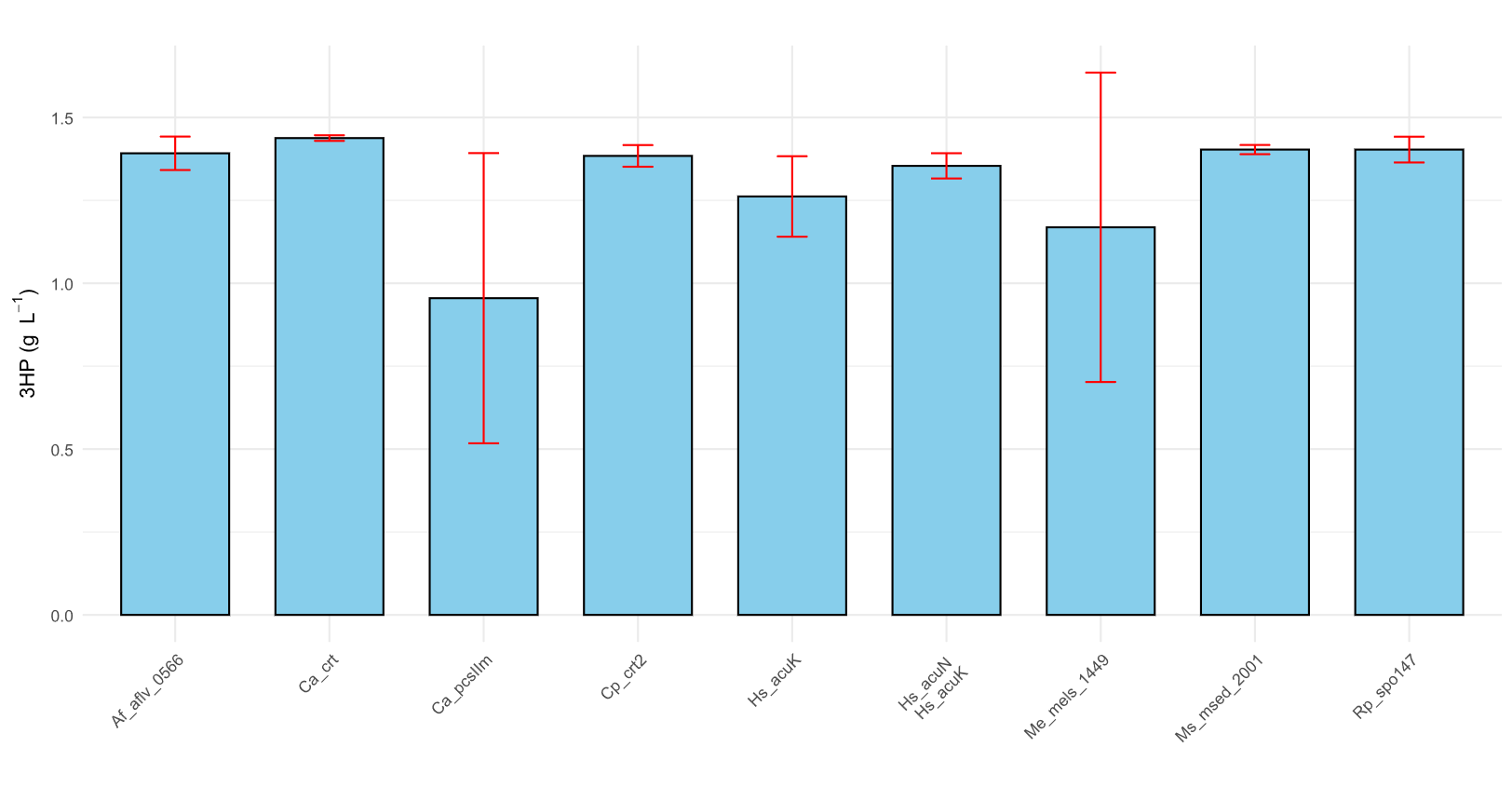


Supplementary Figure 14. 3HP concentration detected at the end of cultivation when screening enzymes dehydrating 3HP-CoA to AA-CoA. The *Hs_acuN*/*Hs_acuK* double gene system employs a 3HP-CoA:acrylate CoA-transferase in contrast to the 3HP-CoA ligase used in the other tested systems. Bar plots represent the mean of n=3 biological replicates, error bars indicate standard deviation.


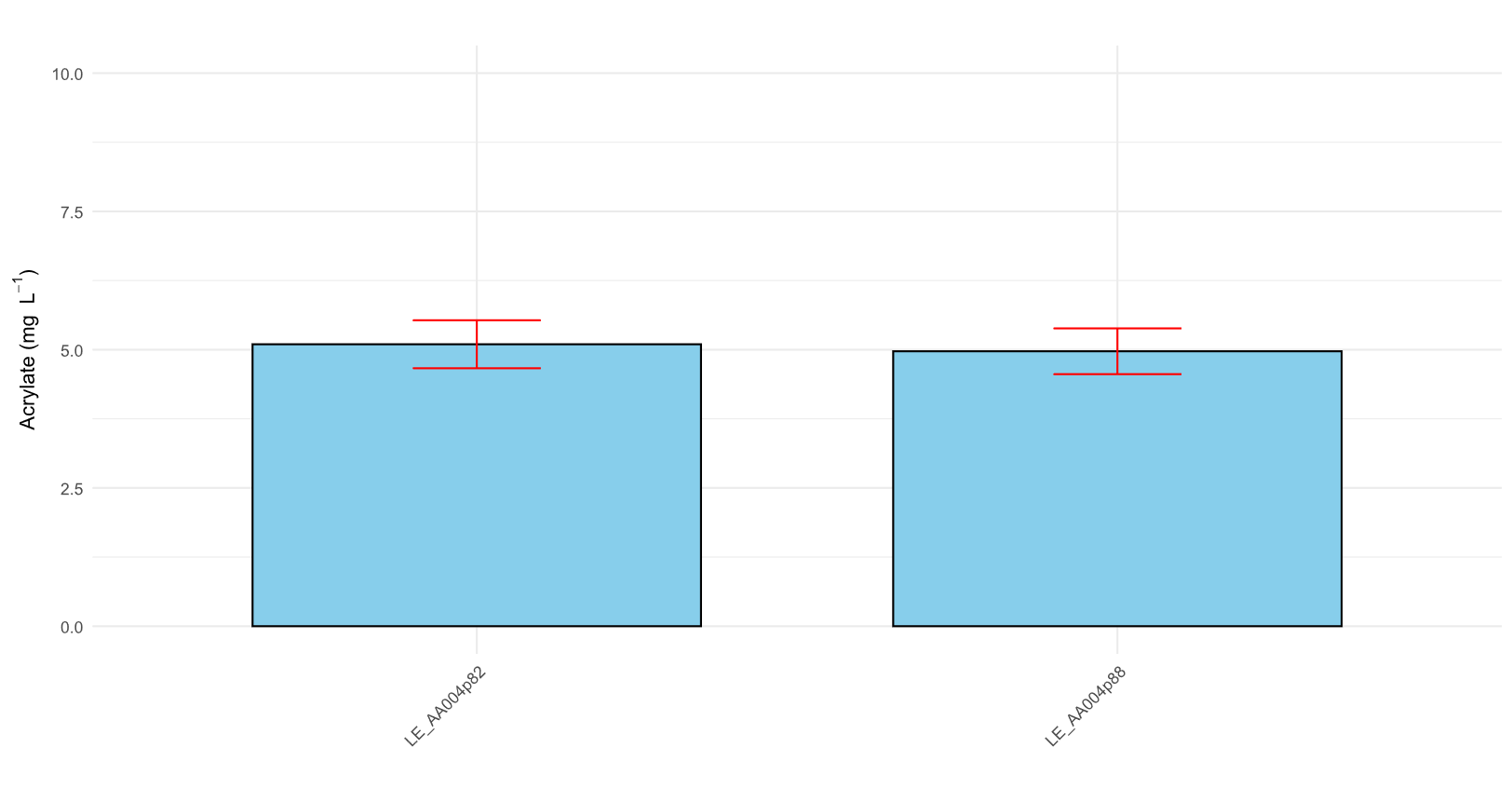


Supplementary Figure 15. AA titre obtained from two 3HP converting plasmids expressed in a non 3HP producing background strain. 3HP was supplemented to the medium at a concentration of 3 g L^-1^. Bar plots represent the mean of n=3 biological replicates, error bars indicate standard deviation.


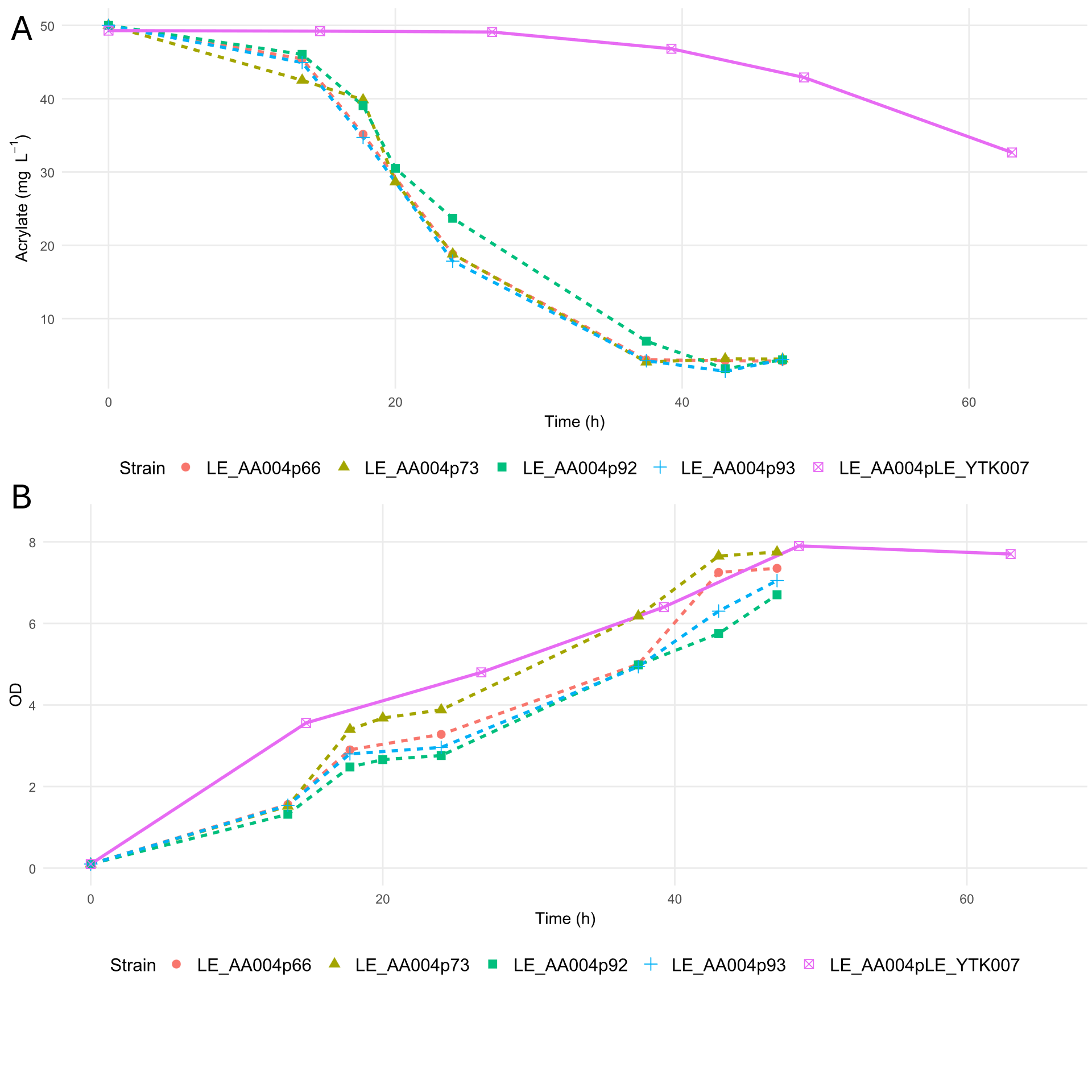


Supplementary Figure 16. (A) Acrylate concentration and (B) optical density (OD_600_) over time for four strains carrying the β-alanine pathway plasmids (p66 to p93) and a control strain carrying the empty vector (pLE_YTK007). Cultures were grown in Delft medium (pH 6.5) supplemented with 50 mg L^-1^ acrylate. Data represent single measurements from individual experiments.


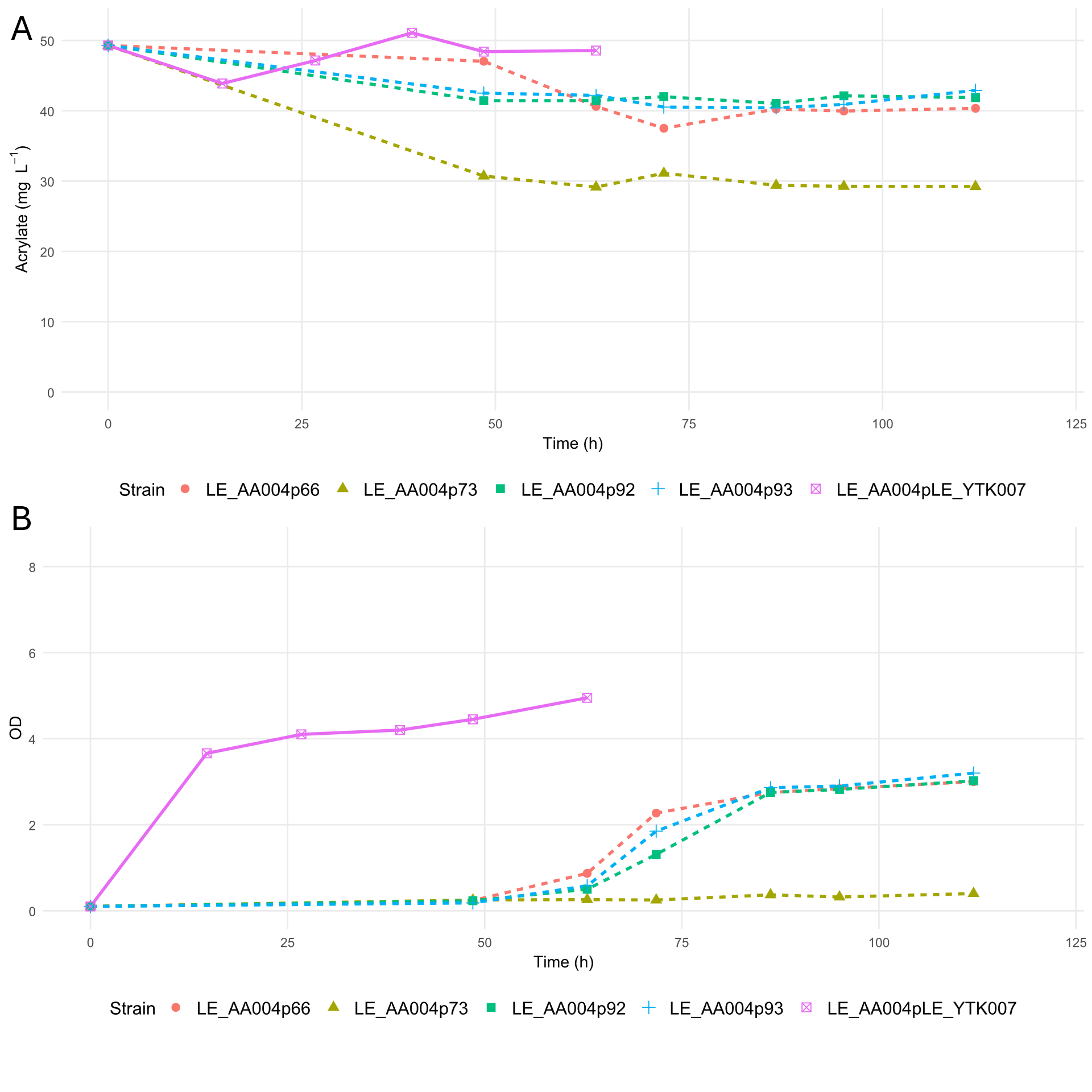


Supplementary Figure 17. (A) Acrylate concentration and (B) optical density (OD_600_) over time for four strains carrying the β-alanine pathway plasmids (p66 to p93) and a control strain carrying the empty vector (pLE_YTK007). Cultures were grown in Delft medium (pH 5) supplemented with 50 mg L^-1^ acrylate. Data represent single measurements from individual experiments.


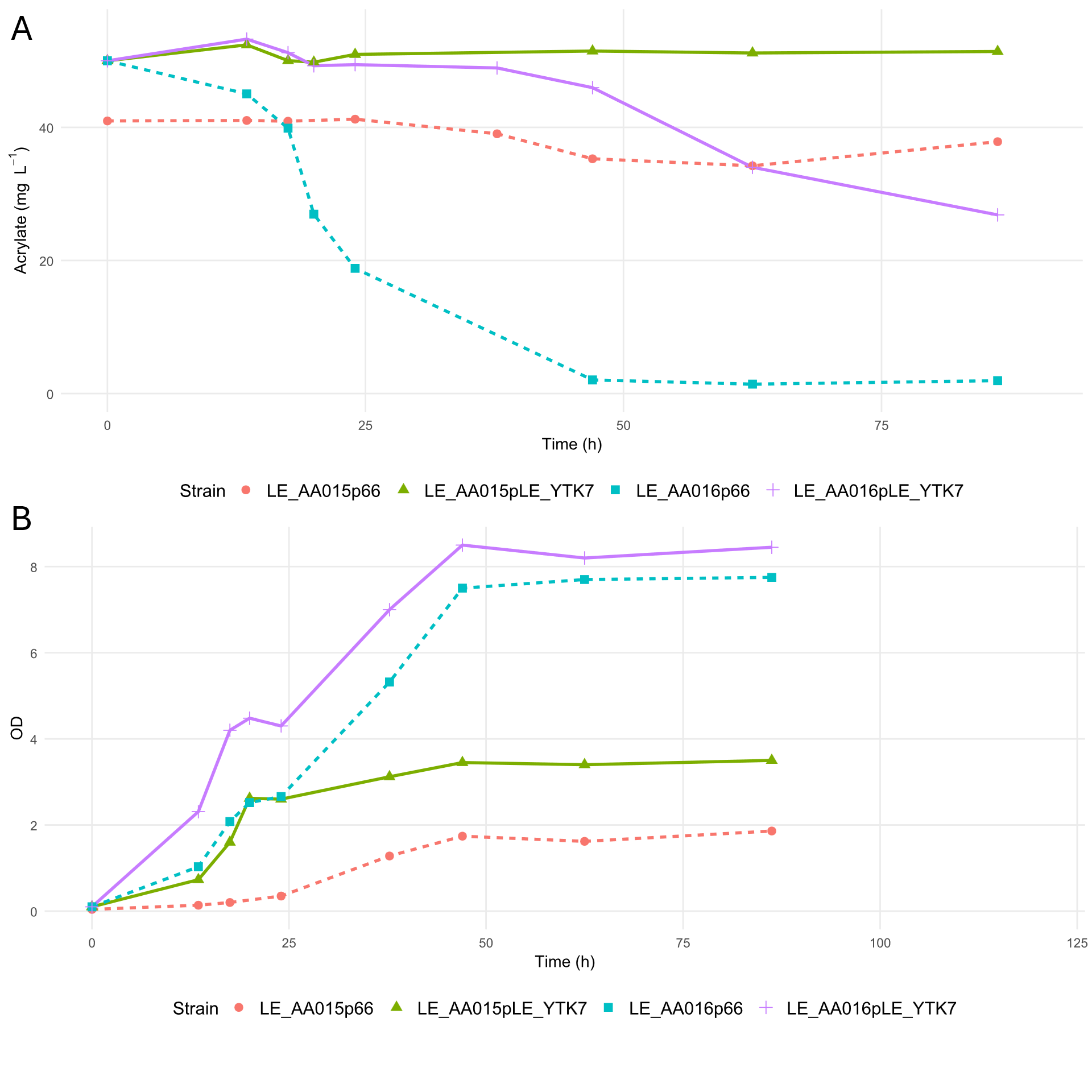


Supplementary Figure 18. (A) Acrylate concentration and (B) optical density (OD_600_) over time for the double mutants ΔACH1 *ETR1*^M1V^ (LE_AA16) and ΔACH1 Δ*ETR1* (LE_AA15) each carrying either the β-alanine pathway plasmid (p66) or the empty vector (pLE_YTK007). Cultures were grown in Delft medium (pH 6.5) supplemented with 50 mg L^-1^ acrylate. Data represent single measurements from individual experiments.


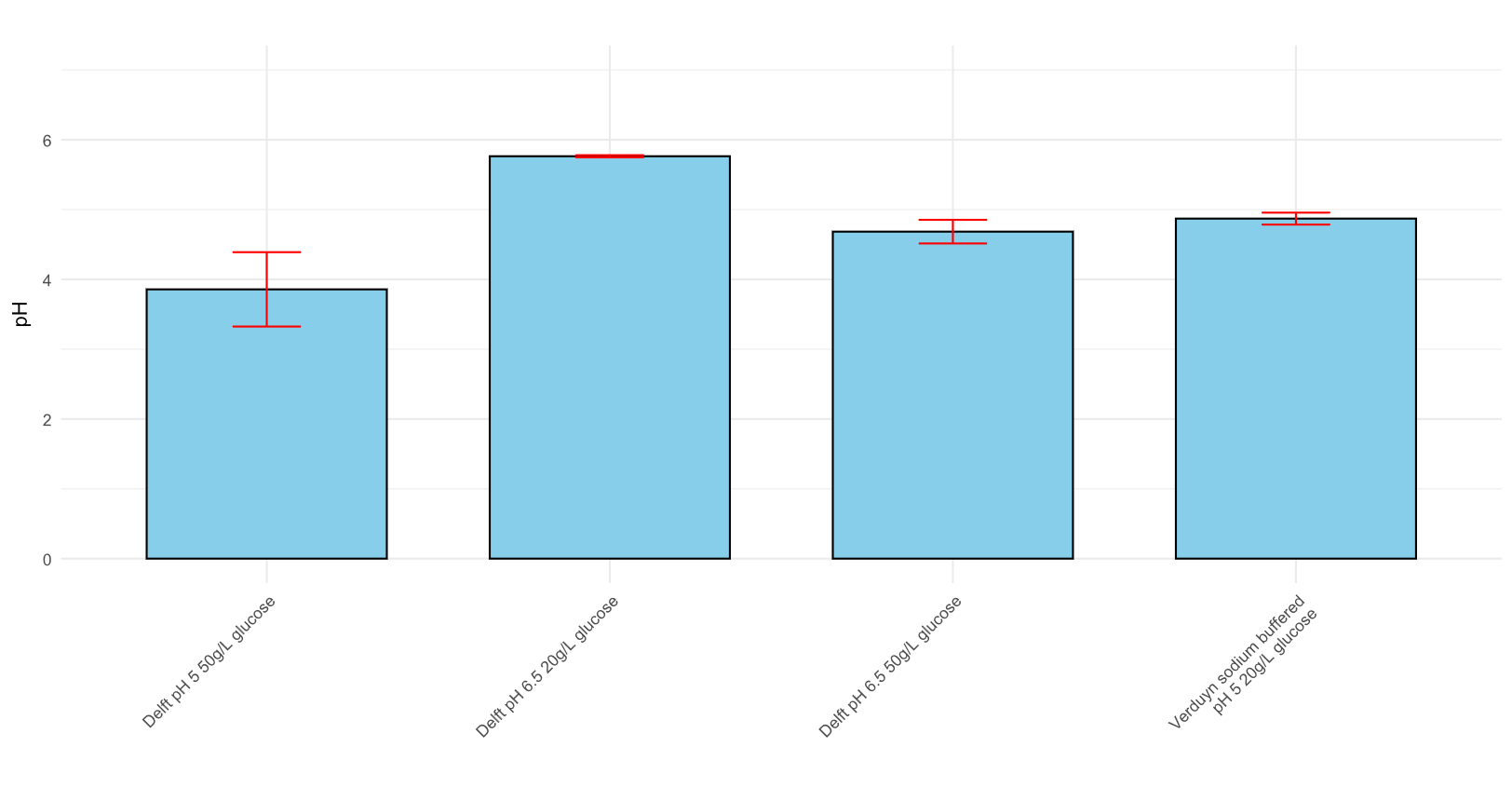


Supplementary Figure 19. pH measured at the end of cultivation for LE_AA004p66 in different media supplemented with 3 g L^-1^ β-alanine. Bar plots represent the mean of n=3 biological replicates, error bars indicate standard deviation. Corresponding AA titres are shown in Figure 4 D.


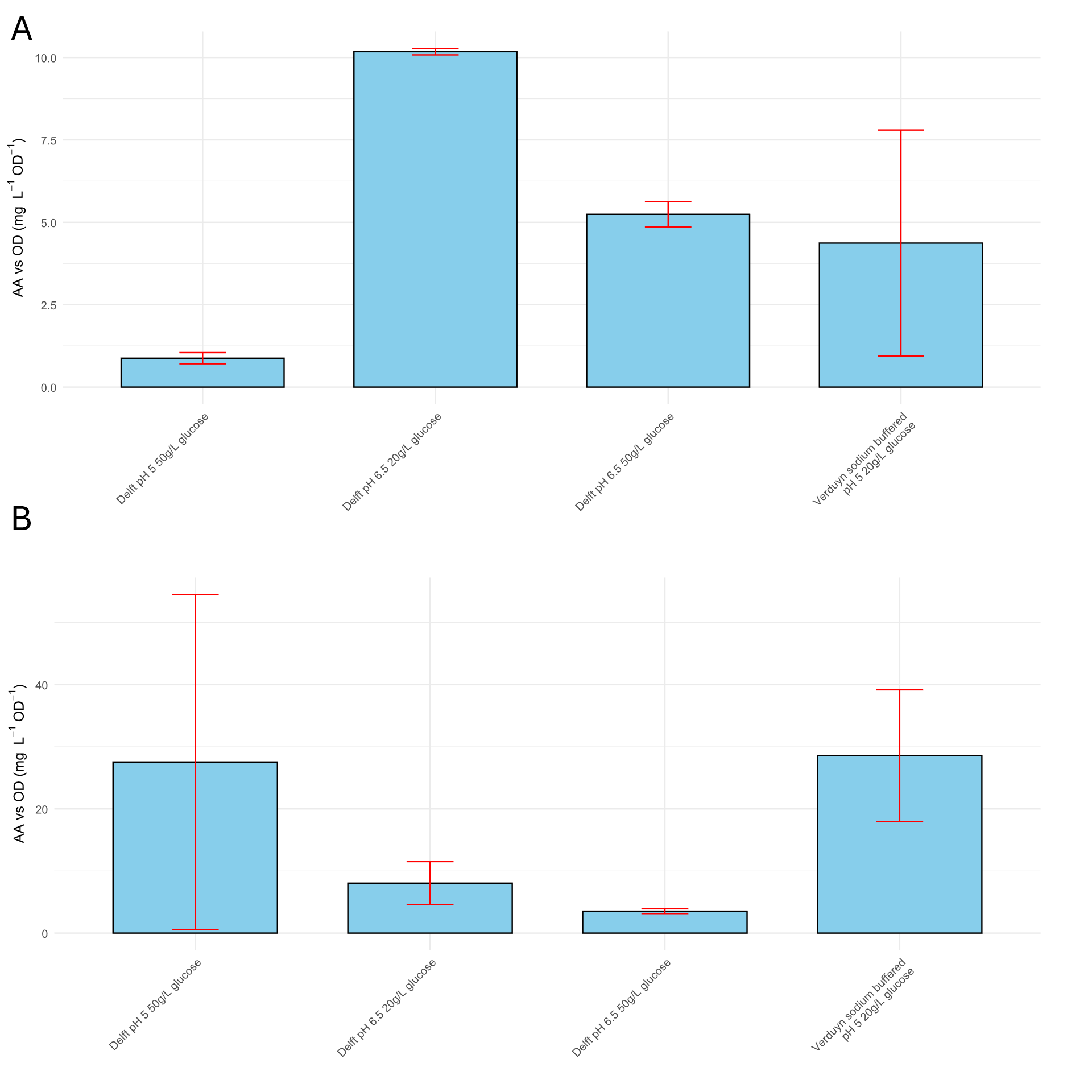


Supplementary Figure 20. AA titres normalised to biomass (OD) in different media supplemented with 3 g L^-1^ β-alanine for (A) LE_AA004p66 and (B) LE_AA015p66. Bar plots represent the mean of n=3 biological replicates, error bars indicate standard deviation. Corresponding AA titres are shown in Figure 4 D.
